# A New Technique for Analysing Interacting Factors Affecting Biodiversity Patterns: Crossed-DPCoA

**DOI:** 10.1371/journal.pone.0054530

**Published:** 2013-01-24

**Authors:** Sandrine Pavoine, Jacques Blondel, Anne B. Dufour, Amandine Gasc, Michael B. Bonsall

**Affiliations:** 1 Muséum national d’Histoire naturelle, Département Ecologie et Gestion de la Biodiversité, UMR CNRS UPMC 7204, Paris, France; 2 Mathematical Ecology Research Group, Department of Zoology, University of Oxford, Oxford, United Kingdom; 3 CEFE/CNRS, Montpellier, France; 4 Université de Lyon, Université Lyon 1, UMR CNRS 5558, Villeurbanne, France; 5 Muséum national d'Histoire naturelle, Département Systématique et Evolution, UMR CNRS 7205, Paris, France; 6 St. Peter’s College, Oxford, United Kingdom; Michigan State University, United States of America

## Abstract

We developed an approach for analysing the effects of two crossed factors A and B on the functional, taxonomic or phylogenetic composition of communities. The methodology, known as crossed-DPCoA, defines a space where species, communities and the levels of the two factors are organised as a set of points. In this space, the Euclidean distance between two species-specific points is a measure of the (functional, taxonomic or phylogenetic) dissimilarity. The communities are positioned at the centroid of their constitutive species; and the levels of two factors at the centroid of the communities associated with them. We develop two versions for crossed-DPCoA, the first one moves the levels of factor B to the centre of the space and analyses the axes of highest variance in the coordinates of the levels of factor A. It is related to previous ordination approaches such as partial canonical correspondence analysis and partial non-symmetrical correspondence analysis. The second version projects all points on the orthogonal complement of the space generated by the principal axes of factor B. This second version should be preferred when there is an a priori suspicion that factor A and B are associated. We apply the two versions of crossed-DPCoA to analyse the phylogenetic composition of Central European and Mediterranean bird communities. Applying crossed-DPCoA on bird communities supports the hypothesis that allopatric speciation processes during the Quaternary occurred in open and patchily distributed landscapes, while the lack of geographic barriers to dispersal among forest habitats may explain the homogeneity of forest bird communities over the whole western Palaearctic. Generalizing several ordination analyses commonly used in ecology, crossed-DPCoA provides an approach for analysing the effects of crossed factors on functional, taxonomic and phylogenetic diversity, environmental and geographic structure of species niches, and more broadly the role of genetics on population structures.

## Introduction

The diversity of a community has traditionally been measured using a variety of simple metrics such as the number of species or the average rarity of species [1]. However, biodiversity is pluralistic [2] and new approaches need to consider how to best integrate differences among species. New methods have recently focused on several kinds of differences among species. These include taxonomic differences (including all taxonomic levels, from species to families and orders) [3], functional differences (be they based on life history, morphological, physiological, ecological or behavioural traits) [4], and, with the advance of molecular techniques, phylogenetic differences [5]. However, whatever aspect of biodiversity is measured (taxonomic, functional or phylogenetic), the aim is to understand diversity across multiple factors. For example, diversity within a region might be explained by the diversity within habitat patches (the so-called alpha diversity) and/or by the differences among habitat patches (beta diversity). A large number of studies have been made using this approach (e.g. [6]–[11]).

One aspect where novel methodologies might be usefully developed is to gain insight in understanding the effect of interacting factors on biodiversity patterns [7,12]. Such factors, often described as crossed factors [13], might be defined by sampling designs in observational or experimental studies. They might focus on spatio-temporal analyses of biodiversity, where for example several regions are sampled at the same period during several successive years, addressing questions such as: can we partition biodiversity across regions and years, and evaluate the marginal effects of space and time? Other crossed factors studied in ecology include the impacts on biodiversity patterns of altitudinal belts × regions (e.g. [14]) and habitats × regions (e.g. the present study).

**Figure 1:**
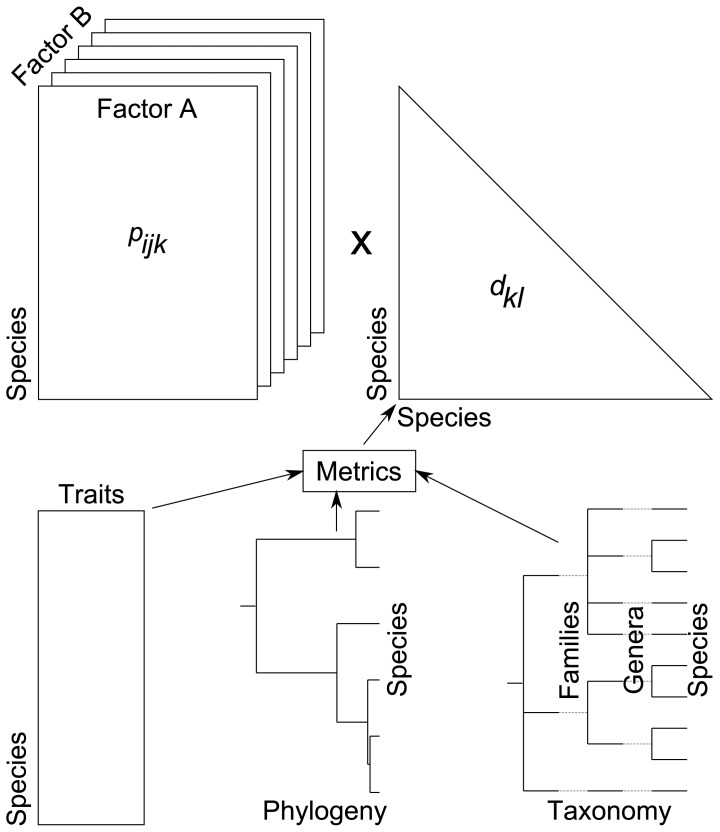
Type of data sets that might be used with crossed-DPCoA. Distance metrics are used to transform raw data (tables of functional traits, phylogenetic trees, taxonomies) into a symmetrical matrix of distances among species.

A popular index of diversity, which is based on proportions and distances, is quadratic entropy [7,12]. Applications of this index in ecology have focussed on species proportions in terms of species-specific relative abundances [9], biomass [15] and distances among species by taxonomic [16], functional [17,18] or phylogenetic metrics [19,20]. Quadratic entropy can thus be used to define any measure of biodiversity (species, taxonomic, functional or phylogenetic). Indeed, at a first level, quadratic entropy is broadly defined as the average distance between two species in a community. At a broader level, it can also be applied to define an average distance between communities based on the species they contain. It can be partitioned among different factors affecting the communities, revealing the separate effects of each of them and any interactions (data structure is given in Fig. 1). Moreover, this index can be used to evaluate and test the strength of the conditional effect of each factor given the other. However, the index provides no explanatory power for understanding this effect (e.g. which levels of the factor are of most influence? which species are involved?).

The objective of this paper is to extend the approach described in [7] and [12], based on quadratic entropy index, with ordination methods, to allow the description of the effects of each factor (in terms of the original species) rather than measuring only the strength of these effects. The rationale of ordination methods is to display and order data on as few axes as necessary to reveal patterns and facilitate their analysis. The methodology we develop is not just another addition to the already long list of ordination approaches. It generalizes several of the most popular ordination analyses including canonical correspondence analysis [21,22] and non-symmetrical correspondence analysis [23,24] (see also [17] for other ordination analyses). It is also more flexible in the analysis of biodiversity patterns, allowing various kinds of data to be processed (e.g. different types of species characteristics including traits, phylogenies or taxonomies; different types of species weights including biomass, densities or abundance). For simplicity, here we focus on the case of two factors affecting biodiversity. In this context, our aim is to use the ordination approach to answer the following questions: if there is a conditional effect on biodiversity of a factor (A) given another factor (B), (i) which levels of factor A exert the greatest influence on biodiversity? (ii) which individual species (or traits, taxonomic levels, clades) are affected by each level within a factor, and in what way? (iii) is the effect of factor A constant under all levels of factor B? (iv) if not, how do the levels of factor B influence the impact of factor A on biodiversity? We present this new methodology and apply it to the analysis of avian phylogenetic diversity across successional forest gradients. Potential applications of the method are reviewed.

## Materials and Methods

### Type of data required to apply the methodology and their preparation

Consider two crossed factors A and B that might affect the diversity of *S* species. Factor A contains *r* levels and factor B contains *m* levels. Communities are defined at the intersection of these two factors: for instance, the community *ij* is the community associated with level *i* (of *r* levels) of factor A and level *j* (of *m* levels) of factor B. We focus here on situations where a single community is associated with each level of factor A and each level of factor B, leading to *rm* communities (but see Text S2 for further discussion on unbalanced schemes and on situations where several plots are associated with each combination of levels of factors A and B).

The basic data needed to characterize the diversity of communities are: (1) the definition of proportions of species within the communities; (2) the definition of how different a species is from another species. Let 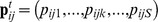, where *t* is the transpose, be the vector of species proportions in the community *ij*. Mathematically, it only needs to satisfy the following properties: 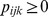 for all *i,j,k* and 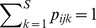. The value *p_ijk_* stands for the proportion of species *k* in community *ij* associated with level *i* of factor A and level *j* of factor B. Biologically, the estimated proportions might be based on density, percentage cover (for plants), biomass or number of individuals. If only presence-absence data are available, our methodology can still be applied by choosing *p_ijk_* = 1/*S_ij_* for all *k*, *i*, *j*, where *S_ij_* is the number of species observed in community *ij*. The choice of the species’ proportions is important and will necessarily affect the result of the analysis. Species with higher proportions in a community are considered to characterize the community better than species with lower proportions. For instance, for organisms with very different biomass, measuring diversity using biomass might be more relevant than using the number of individuals (e.g., [25]). When the objective is to compare the composition of communities, species with high and unequal proportions over all communities will contribute more to the definition of the differences among communities than species with low, even if unequal, proportions in all communities. Ecological data fundamentally contain a strong imbalance in species’ proportions, especially when they are measured based on relative number of individuals. Simple transformations (e.g. square root) of the species biomass, percentage cover (plants) or abundance might be considered before the ultimate transformation into proportions to avoid there being an overwhelming influence of only a few species on the result of the diversity analysis.

The dissimilarities among species must be defined and incorporated into a 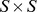 matrix 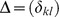, where *S* is the number of species. Such dissimilarities can be obtained for example from taxonomies (leading to the analysis of taxonomic diversity), phylogenies (leading to phylogenetic diversity), or biological traits (e.g. morphological, life-history, behavioural traits leading to functional diversity). However, we do require that **Δ** be Euclidean, that is to say, *S* points can be embedded in a Euclidean space such that the Euclidean distance between points *k* and *l* (ordinary distance between two points) is *δ_kl_*. Phylogenetic dissimilarities defined as the square root of the sum of branch lengths (or number of nodes) in the shortest path that connects the two species on the phylogenetic tree satisfy these conditions [26]. Taxonomic dissimilarities with Euclidean properties can be obtained as follows: the dissimilarity between two species in the same genus is 1, the dissimilarity between two species in the same family but not the same genera is 2, and so on [27]. The metrics available to transform a set of biological or functional traits into a matrix of functional dissimilarity among species are numerous and depend on the types of data associated with the traits being considered (i.e. nominal, quantitative, binary, etc). Metrics that fulfil the above conditions of being Euclidean can be found for instance in [28] and [29].

To summarize, the approach starts with *S* species, *rm* communities, vectors 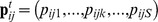 of species’ proportions within communities *ij* for all *i* and *j*, a 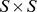 matrix 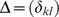 with *δ_kl_* a measure of dissimilarity between species *k* and *l* with Euclidean properties, and two crossed factors that describe the communities. Hereafter, we will also consider:  the vector of species proportions, 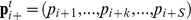, associated with level *i* of factor A, 
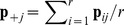
 the vector of species proportions, 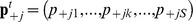, associated with level *j* of factor B, and 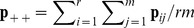 the vector of species proportions, 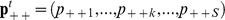, over the whole data set.

### The space of double principal coordinate analysis-basics

The method ‘double principal coordinate analysis’ (DPCoA) was developed by Pavoine et al. [17] to compare several communities containing species that differ according to their taxonomic, morphological or biological features. A key step of this approach is the definition of a common Euclidean space that embeds both species and communities. To obtain this common space, a principal coordinate analysis (PCoA) is first applied to species distances (**Δ**) where each species *k* is weighted by its global proportion 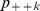 [30]. The PCoA of **Δ** generates a cloud of points in a geometric (Euclidean) space of orthogonal axes, where each point represents a species. The space is defined by axes called principal axes. The coordinates of the species on the principal axes are given by the rows of a 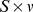 matrix (**X**), where 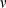 is the number of principal axes and thus the dimension of the space. With each principal axis is associated a value, named an eigenvalue, that measures the variance of the species' coordinates along that axis where species are weighted by their global proportions 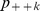. The first axis is in the direction of highest possible variance; the second axis is perpendicular to the first one and is in the direction of second maximum variance; and so on. The first axes of the space thus optimize the representation of the dissimilarities among species in few dimensions. Let *M_k_* define the point that corresponds to species *k* in the full *ν*-dimensional PCoA space. By definition of PCoA, 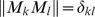 for all *k* and *l*, where 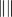 designates the Euclidean distance between two points. A well-known measure of point dispersal, referred to as inertia, is defined as
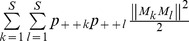
(1)


This inertia is equal to the sum of all eigenvalues.

The communities are positioned in this space at the centroids of the species they contain (centroids are defined in terms of means of species’ coordinates on the principal axes; details are given in Text S1 Proof 1). Consider the 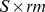 matrix 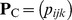 with species as rows and communities as columns. The coordinates of the communities are given in the rows of matrix 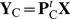. Let *C_ij_* define the point that corresponds to community *ij*, then the inertia of communities’ points is 
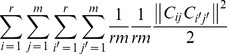
(2)


More generally, the dispersion of species’ and communities’ points in this space provides a geometric representation of the decomposition of quadratic entropy (QE) given by Rao [31]. QE can be defined as [32,33] 
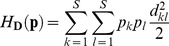
(3)


where **D** = (*d_kl_*) is a matrix of pairwise dissimilarities and 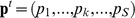 is a vector of proportions. This QE index gives high weights to the highest proportions [34–36]. With the notations given in the previous section, according to Rao, the total diversity over all communities is 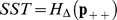, i.e. the inertia of species’ points in the space of DPCoA (eqn 1).

Consider two proportion vectors 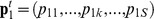 and 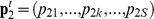, and **D** = (*d_kl_*) a matrix of pairwise dissimilarity; Rao [31] also defined a cross-entropy index between two vectors of proportions as:
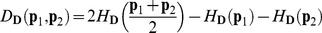
(4)


where



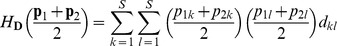
.

It has been shown that 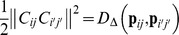 for all *i*, *i*’, *j* and *j*’ (proof in [17] and Text S1). The name double PCoA thus stems from the fact that both the dissimilarities among species and the dissimilarities among communities, sensu Rao [31], are embedded in a Euclidean space. Let 
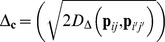
 be the matrix of pairwise dissimilarity among communities and 
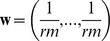
 be the *rm* × 1 vector of communities’ weights (even weights). According to Rao [31], a component of diversity among communities is 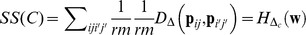, i.e. the inertia of communities’ points in the space of DPCoA (eqn 2). Then, the total diversity over all communities (*SST*) is equal to the sum of the component of diversity among communities (*SS(C)*) and *SSW*, a component of diversity within communities (
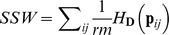
).

### The space of DPCoA-extension

Next, consider that communities are defined according to two crossed factors. Attributes of factors A and B will thus also be positioned in the space of the DPCoA. We define the following matrices of species proportions: the 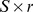 matrix 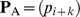 with species as rows and levels of factor A as columns; the 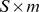 matrix 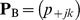 with species as rows and levels of factor B as columns (the notations were given in section *Type of data required to apply the methodology and their preparation*). In the space of the DPCoA, the coordinates of the levels of factor A and the levels of factor B, respectively, are given in the rows of the following matrices: 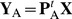, 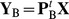. Similarly as a community *ij* was placed at the centroid (mean of species’ coordinates per axis) of the species’ points weighted by values of **p** *_ij_* (that gives each species’ importance in the community *ij*), an attribute *i* of factor A, for example, is positioned at the centroid of species’ points weighted by values of **p** *_i+_* (giving each species’ importance over all communities associated with attribute *i* of factor A). Let *A_i_* define the point that corresponds to the *i*th level of factor A, *B_j_* define the point that corresponds to the *j*th level of factor B. It can be shown that 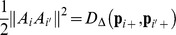, 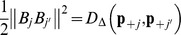, for all *i*, *i*’, *j* and *j*’ (proof in Text S1). This means that the half squared Euclidean distance between the positions of two levels of a factor is simply the function of dissimilarity between two vectors of proportions developed independently by Rao [31] and also used to compute dissimilarities among communities.

The inertia of points in this space can thus be associated with a partitioning approach of the index QE (e.g. [37]): the analysis of quadratic entropy (ANOQE). Compared to the previous section, the crossed-factors will now affect the partitioning of the index QE. ANOQE is an ANOVA-like approach where the measure of variance is replaced with quadratic entropy [38]. ANOQE is the application to quadratic entropy of a more general approach, named analysis of diversity (ANODIV), which can be applied to any diversity indices at least satisfying the property of concavity (i.e. diversity increases by mixing) (e.g. [7,39]).

Let 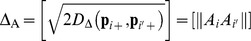, where 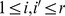, be the matrix of dissimilarity between the levels of A and 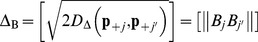, where 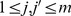, be the matrix of dissimilarity between the levels of B. The diversity partitioning given in the previous section is complemented by the fact that the component of diversity among communities *SS*(C) is equal to the sum of the diversity related to factor A (main effect, and inertia of points associated with levels of factor A) 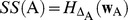



where 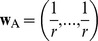 is the vector of weights attributed to each level of factor A (here even weights but see Text S1 and Text S2 for alternatives), plus the diversity related to factor B (main effect, and inertia of points associated with levels of factor B)
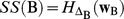



where 
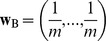
 is the vector of weights attributed to each level of factor B (here even weights but see Text S1 and Text S2 for alternatives), plus the diversity due to the interaction of the factors A and B



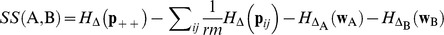
.

This leads to
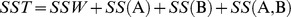



A simpler expression for the component *SS*(A,B) of interaction can also be obtained. Let *Σ_ij_* be a point located at coordinates (**p** *_ij_*−**p** *_i_*_+_−**p**_+*j*_+**p**_++_)*^t^* **X**. This point represents the position community *ij* would have if all positions of the levels of factor A and those of the levels of factor B were moved to the centre of the space of DPCoA. This re-centring process would remove the main effects of A and B. With these notations, the inertia of points *Σ_ij_* for all *i* and *j* would be (Proof in Text S1)
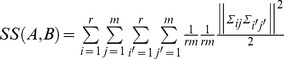



With 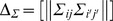 and given that 
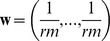
 is the *rm* × 1 vector of communities’ weights (even weights), 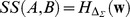.

### Crossed DPCoA

Now the aim of crossed-DPCoA is to visualize the pattern of diversity due to factor A knowing the existence of a crossed factor B (the conditional effect of factor A given B); and the analysis of factor B knowing factor A can be obtained by a similar approach. Several methods allow the analysis of two crossed factors (e.g. [40,41]). We explore two of them below (further discussion can be found in Text S2 and S7).

The crossed-DPCoA approach starts from the space of DPCoA where species, communities, attributes of factor A and attributes of factor B are displayed by points (see the two previous sections). The axes of this space best represent the distances among species points, so that the first axes of the space provide a representation in few dimensions that summarize the main patterns in the (taxonomic, phylogenetic or functional) distances among species. To analyse the main effect of one of the factors, say factor B, it is sufficient to determine the principal axes of the positions of the levels of this factor (axes of highest variance in the coordinates of the levels of factor B instead of the coordinates of the species). The points for the species and levels of factor B defined in the space of DPCoA are projected onto these principal axes, regardless of the other points. This corresponds to applying DPCoA to 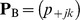, the matrix of proportions of species associated with levels of factor B, and **Δ**, the matrix of dissimilarities among species. Here we go one step further by taking the second factor into account.

To visualize the pattern of diversity due to factor A knowing the existence of a crossed factor B, we need to project all points into a new space, the axes of which best differentiate communities, thanks to their association with attributes of factor A. When defining these new axes, we also need to control for factor B. The methods are described below and their mathematics is detailed in Text S2.

### Version 1: Mean-based approach

This first version aims at moving the positions of all levels of factor B at the centre of the space to remove the amount of diversity among communities due to the main effect of B. This first version should always be performed, even if eventually version 2 is used to provide complementary information on the effects of factor A relative to B. It depends on the average effects of factor B on community compositions only. A useful property of the space of DPCoA is that all clouds of points are centred: 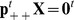, 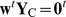, 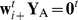, 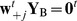, where **0** is the vector of zeros of appropriate size (Proofs in Text S1). Another useful property is that the position of a level *i* of factor A is at the centroid of communities’ points for that level: 
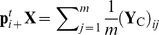
, where 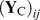 is the coordinates of community *ij*. Similarly, the position of a level *j* of factor B is at the centroid of communities’ points for that level: 
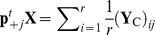
. To eliminate the main effect of B on the positions of the communities, the positions of the levels of B are moved to the centre of the space. Although the positions of the species are unchanged, the communities are driven in this displacement. The coordinates of the communities are thus re-centred and are given by the rows of matrix 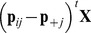 instead of matrix 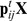. Let (**Y**_B_)*_j_* be the vector of coordinates for level *j* of factor B in the space of the DPCoA. The coordinates of the community *ij* in the re-centred space are thus (**Y**_C_)*_ij_*−(**Y**_B_)*_j_*. The vector of coordinates of level *i* of factor A is 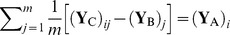, where (**Y**_A_)*_i_* is the coordinate of level *i* of factor A in the space of DPCoA. The positions of the levels of factor A are thus unchanged by the centring process. The inertia of the new communities’ points becomes *SS*(C)−*SS*(B) = *SS*(A)+*SS*(A,B), which is the total effect of A (Text S2). The inertia of the positions of the levels of factor A remains *SS*(A). The last step of crossed-DPCoA-version 1-consists in analysing the principal axes of the points that locate the levels of factor A (axes of highest variance in the coordinates of the levels of factor A; sum of eigenvalues of these axes equals *SS*(A)) and in projecting on these axes the positions of the species and the new positions of the communities.

### Version 2: Structure-based approach

The first version above consists in moving centroids (defined by the levels of factor B) into the centre of the space of DPCoA. Version 2 developed here should be used, in complement to version 1, when the factor A is known a priori to present some correlation with factor B. Version 1 and version 2 will give the same effective result if the two factors are operating in orthogonal directions. Instead of solely removing the main effect of B by only moving centroids, we can think of removing the effect of B by projecting all points in the orthogonal complement to the subspace generated by these centroids. This process will move the positions of the levels of factor B to the centre of the new space, as in version 1 of crossed-DPCoA, and it will, in addition, eliminate any diversity patterns due to factor A only or to the interactions A×B that are in the same direction as that of the diversity pattern generated by factor B. Thus, only that part of A which is fully independent of the main effect of factor B will remain. Let G_X_ be the space generated by the species’ points, G_B_ the space generated by the points associated with the levels of factor B, and 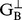 the orthogonal complement to G_B_, then G_X_ =  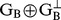. In subspace 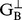, the inertia of levels of factor A is lower than *SS*(A); the inertia of communities’ points is lower than *SS*(A)+*SS*(A,B) (See Text S2 for simple examples of projections in subspace 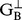). This analysis is possible only if the number of principal axes of species’ points (dimension of G_X_) is higher than the number of principal axes of the points of the levels of factor B (dimension of G_B_). Otherwise the subspace 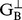 would be empty. All points are projected into 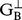. Then the principal axes of the new positions of the level of factor A in 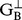 (axes of highest variance in the coordinates of the levels of factor A) are defined and all points are projected on the final subspace generated by these principal axes.

### Case study

We applied the above two versions of crossed-DPCoA to investigate whether there is phylogenetic convergence in avian communities along successional forest gradients [42,43]. Five locations were considered: three in the Mediterranean region (Provence, southern France; Corsica Island, southern France; and north east Algeria) and two in the central European region (Burgundy, central France; and Poland). In each location, a habitat gradient has been conventionally divided into six seral stages (intermediate stages found in forest ecosystems advancing towards their climax stage after a disturbance event) in such a way that all five selected habitat gradients match one another reasonably well in terms of the number, patterns and overall structure of habitats. Selection of habitats was made using classical criteria of habitat patterns, especially the complexity and height of the vegetation (ranging from low bushy vegetation, less than 1 m height (stage 1), to forests with trees at least 20 m high (stage 6)). The density of bird species has been determined in each location and each habitat stage (see Blondel and Farré [42] for further details on the methodology). A composite phylogenetic tree was obtained based on Davis’ supertree [44] that is a strict consensus of 2000 trees (see details in Text S5). Pairwise phylogenetic distances between species were simply defined as the number of edges on the smallest path that connects them on the phylogenetic tree. The two versions of crossed-DPCoA were applied to analyse the effects of differences among locations (factor A) given the habitat stage (factor B). The R script is available in Text S3; a manual is given in Text S4; and data are available in Dataset S1.

## Results

**Figure 2:**
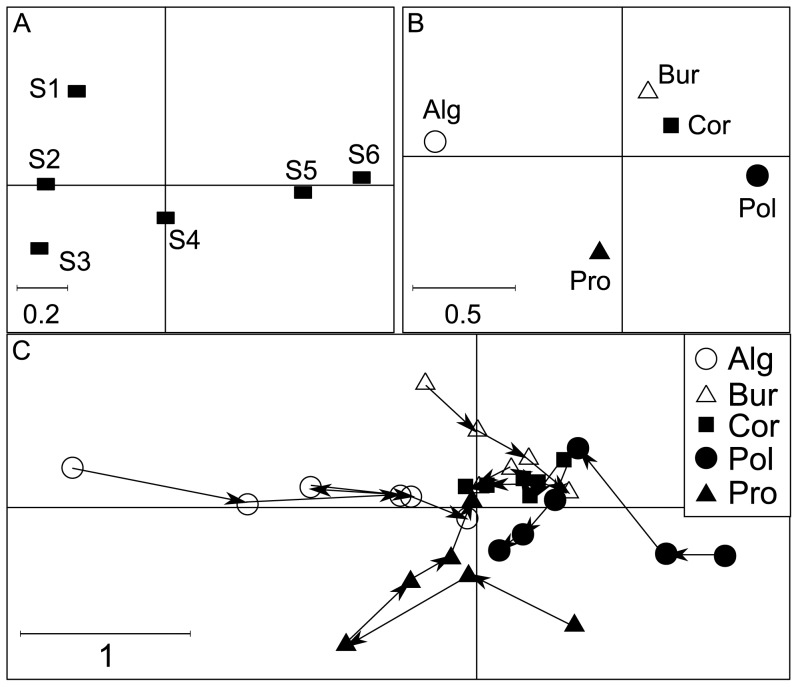
Preliminary analysis of factor B (DPCoA) and results of crossed-DPCoA version 1 of factor A given B. (A) Coordinates of the levels of factor B (habitat stages) on their first (horizontal) and second (vertical) principal axes from S1 (first, open habitat stage) to S6 (last, forested habitat stage). (B) Coordinates of the levels of factor A (locations) on the first two axes of crossed-DPCoA version 1. Alg  =  Algeria, Bur  =  Burgundy, Cor  =  Corsica island, Pro  =  Provence, Pol  =  Poland. (C) Positions of the communities, i.e. of the locations given each habitat stage, on the first two axes of crossed-DPCoA version 1. For each location, the arrows connect the habitat stages from S1, open, to S6, closed, forested habitat.

**Figure 3:**
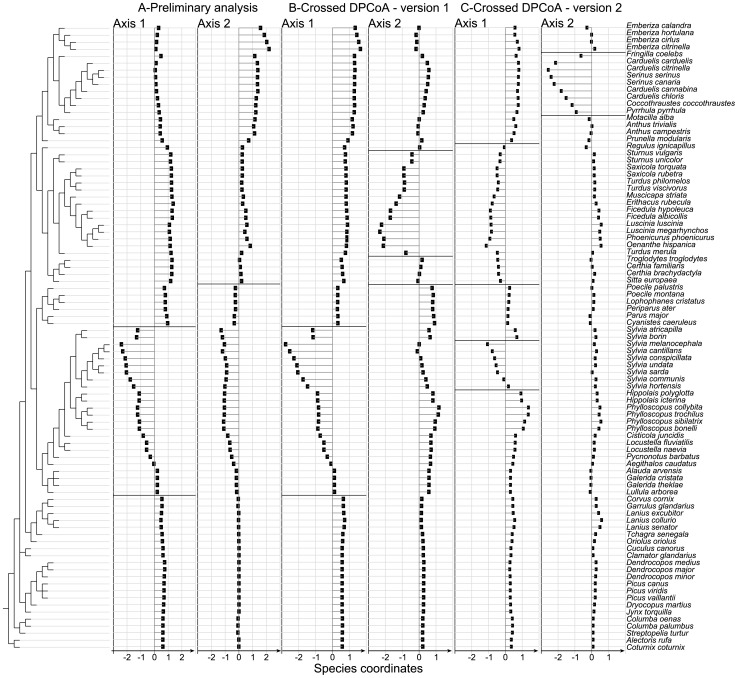
Coordinates of species in crossed-DPCoA. (A) preliminary analysis: first two principal axes of factor B (habitat stages) in the DPCoA stage; (B) first two axes of crossed-DPCoA version 1; (C) first two axes of crossed-DPCoA version 2. The signs of species coordinates are important because they are related to the signs of the coordinates of factor levels in Fig. 2 and 4 as shown in the main text. Horizontal thick lines delimitate groups of related species with the similar sign of their coordinates. The taxonomy used to describe patterns in the coordinates of the species in the main text is given in Text S6.

As both versions of crossed-DPCoA search to eliminate the main effect of factor B, we first analysed this main effect by defining the principal axes of the positions of the levels of factor B in the space of the DPCoA. This approach corresponds to DPCoA applied to the matrix with species as rows, levels of factor B as columns, and densities as entries, and to the matrix of phylogenetic distances among species. The first two principal axes expressed 84% and 12% of the main effect of B, i.e. *SS*(B), respectively. The first axis discriminated open habitats (first three stages of the successional gradient) on the negative side from the most forested habitats, stage 5 and 6, on the positive side, with stage 4 having an intermediate position (Fig. 2A). We interpret the positions of the species by grouping them into families. The list of families and a full taxonomy is given in Text S6. Because they had negative coordinates on the first principal axis of the levels of factor B, we can deduce that the species that characterized, by their higher proportions, the open habitats are the Sylviidae, Acrocephalidae, Phylloscopidae, and related species (Fig. 3A, axis 1). Because they had positive coordinates on the first axis, the species that characterized, by their higher proportions, the forested habitats are the Turdidae, Muscicapidae and related species (Fig. 3A, axis 1). The second axis distinguished stage 1 on the positive side from stage 3 on the negative side with stage 2 having an intermediate position (Fig. 2A). Because they had positive coordinates on the second axis, Emberizidae and Fringillidae generally had higher proportions in stage 1 compared to Sylviidae, Acrocephalidae, Phylloscopidae and related species (with negative coordinates on the second axis) that retained the highest relative densities in stage 3 (Fig. 3A, axis2).

### Mean-based approach (Version 1 of crossed-DPCoA)

The first and second axes respectively of this version of crossed-DPCoA expressed 63% and 17% of the variance in the position of the levels of factor A. All regions were distinguished on these two axes except Corsica, for which all communities were close to the centre of the map (Fig. 2B,C). The first axis mainly distinguished Poland with a positive coordinate from Algeria with a negative coordinate, particularly in the first vegetation stage (open habitat) (Fig. 2B,C). The successional gradient was clear, as the communities became more and more similar in the forested habitats (which can be seen by the fact that the positions of all regions in forested stages are very close to one another and in the centre of Fig. 2C). We know that crossed-DPCoA–version 1–eliminates the average differences among vegetation stages (centroids) but not the subspace generated by these centroids. Indeed, the coordinates of the species on the first two principal axes of factor B (Fig. 3A) were very similar to the coordinates of the species on the first axis of crossed-DPCoA version 1 (Fig. 3B). Communities located on the positive side of the first axis of crossed-DPCoA version 1 (Fig. 3B, axis1), such as the first two vegetation stages for Poland, contained high proportions of species which are characteristic of both sides of the successional gradient: stage 1 with Emberizidae and Fringillidae (located on the positive side of the second principal axis of factor B, Fig. 2A, axis 2) and stages 5 and 6 with Turdidae and Muscicapidae and related species (located on the positive side of the first principal axis of factor B, Fig. 2A, axis 1), and to a lesser extent (due to lower densities of the species of these clades in our data set) the most basal clades located on the bottom of Fig. 3 especially the species *Lanius collurio* (Fig. 3A, B). In contrast, communities located on the negative side of the first axis of crossed-DPCoA version 1 (Fig. 3B, axis1), mostly the first stages for Algeria, contained species which are characteristic of all the first three vegetation stages, such as many Sylviidae, some Acrocephalidae and Phylloscopidae (located on the positive side of the first principal axis of factor B, Fig. 2A, axis 1). The differences between Algeria and Poland were driven by the high proportion for *Sylvia melanocephala* in the first stage of Algeria and the high proportion for *Emberiza citrinella* in the first stage of Poland (Fig. 3B axis 1 and Dataset S1).

### Structure-based approach (Version 2 of crossed-DPCoA)

**Figure 4:**
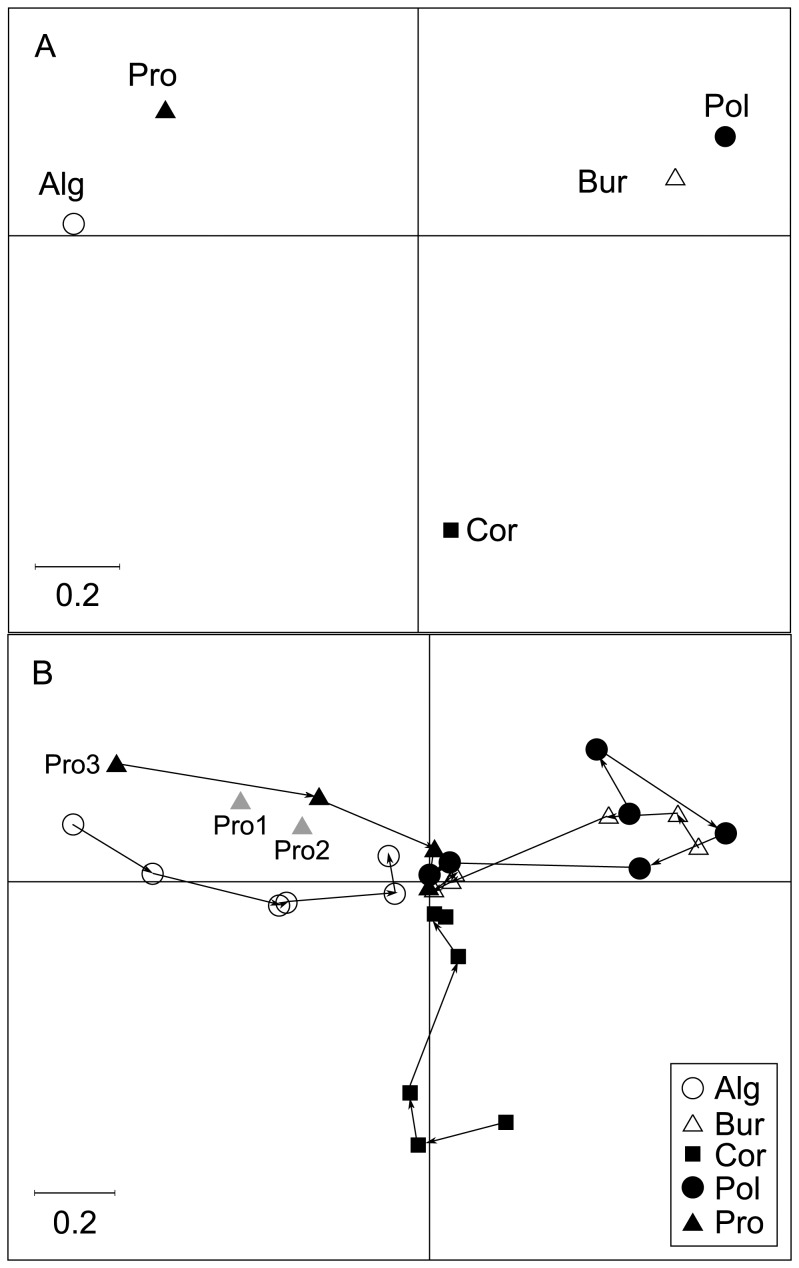
Result of crossed-DPCoA version 2. (A) Coordinates of the levels of factor A (locations) on the first (horizontal) and second (vertical) axes of crossed-DPCoA version 2. Alg  =  Algeria, Bur  =  Burgundy, Cor  =  Corsica Island, Pro  =  Provence, Pol  =  Poland. (B) Positions of the communities, i.e. of the locations given each habitat stage, on the first two axes of crossed-DPCoA version 2. For each location, the arrows connect the habitat stages from S1, open, to S6, closed, forested habitat.

The first axis of this structure-based approach to crossed-DPCoA revealed a contrast between Provence and Algeria (Mediterranean communities with negative coordinates), and Burgundy and Poland (central European communities with positive coordinates), with Corsica having an intermediate position and being distinguished on the negative side of the second axis of crossed-DPCoA version 2 (Fig. 4A). The differences between the pairs of locations Provence-Algeria and Burgundy-Poland observed on the first axis depended on groups of related bird species identified from Fig. 3C. The most characteristic species of Poland and Burgundy (in comparison to other locations) are the *Phylloscopus* species with positive coordinates on the first axis of crossed-DPCoA version 2 (Fig. 3C). In contrast, the Muscicapidae, Turdidae and other species (from *Sturnus vulgaris* to *Sitta europaea* in Fig. 3C, axis 1) and *Sylvia* species (except *Sylvia atricapilla and S. borin* in Fig. 3C, axis 1), with negative coordinates on the first axis of crossed-DPCoA version 2, are more characteristic of Algeria and Provence. Crossed-DPCoA, on the second axis, also clearly highlighted the higher relative abundance of Fringillidae species in Corsica (from *Carduelis carduelis* to *Pyrrhula pyrrhula*, with negative coordinates in Fig. 3C, axis 2).

This phylogenetic structure changes according to each habitat stage. The phylogenetic differences among locations decrease with the habitat stage and reach a point of minimal difference in the sixth seral stage which corresponds to forested habitats (Fig. 4B). The successional gradient thus remained within the communities that became more and more similar in the forested habitats (which can be seen by the fact that the positions of all regions in forested stages are very close on Fig. 4B).

## Discussion

In this study, we have developed a crossed-DPCoA approach and applied it to investigate the effects of phylogenetic diversity on avian community structure. In this section, we discuss the implication of the method for understanding the effects of phylogenetic changes in avian communities along habitat gradients, i.e. ecological successions. Then we consider more generally the impact that crossed-DPCoA could have in and beyond ecological studies.

### Phylogenetic similarities among forest habitats in central European and Mediterranean avian communities

Phylogenetic similarities in avian communities are high in forested habitats of geographically distant locations of Europe and the Mediterranean region. Using species composition measures, Blondel and Farré [42] found that European and Mediterranean locations tend to share more species in closed forest habitats than in open bushy habitats. Our approach, using both versions of crossed-DPCoA, demonstrates that this increased similarity in the composition of different geographically distant forest habitats also stands for the phylogenetic composition of bird communities: even if two forest habitats do not share all their species they have closely related species (belonging to the same genera or families). Most of the phylogenetic differences among the locations occur in open habitats associated with early stages of succession.

The first version of the analysis mostly shows differences between Algeria and Poland and these differences are related to the same species as those that characterize the average differences among habitats: species with the highest relative densities in Algeria are characteristic of open habitats from stage 1 to stage 3 of the successional gradient, especially *Sylvia melanocephala*, whereas species with the highest relative densities in Poland are characteristic of both extremities of the successional gradient, especially *Emberiza citrinella* in the first stage of Poland, and Muscicapidae in stages 5 and 6. These differences between Algeria and Poland might be due to the low species richness observed in the first stages at these locations (dataset S1). The first vegetation stage in Algeria and Poland are dominated each by a single species, *Sylvia melanocephala* and *Emberiza citrinella*, respectively. They are not very diverse. In contrast all other regions exhibited higher species richness and evenness in species densities throughout the succession (dataset S1).

Eliminating the species space that characterizes the average differences among habitats, the second version of the analysis shows differences between the continental Mediterranean locations (represented by Sylviidae species (except *Sylvia atricapilla* and *S. borin*), Muscicapidae and phylogenetically associated species) and the central European locations (represented by Emberizidae, Fringillidae, Motacillidae, Phylloscopidae and a few other non-passerine species). *Sylvia atricapilla* and *S. borin* are the only not exclusively Mediterranean species of the genus. They differentiated much earlier than the other *Sylvia* species, ca 6.5 Ma [45]. This discrimination between the Mediterranean *versus* central European bird communities is in line with previous studies [42] which showed that forest bird biota (that occupy the later successional stages along the habitat gradient) are homogeneous over the whole western Palaearctic, whereas bird communities differ significantly in open habitats where they are much more region-specific (and affected by early stages of the habitat succession). As such, bird communities are expected to differ in the early stages of successions compared to those communities found in old successional habitats. Explaining this pattern requires more attention to the dynamics of landscapes that affected speciation processes, particularly those processes acting over longer geological time scales: from the late Pliocene and thorough the Quaternary when most extant bird species differentiated. Isolation of habitats, which is a prerequisite for allopatric speciation in birds, is likely to have occurred in open and patchily distributed landscapes and this may explain the distribution of avian communities at the regional, landscape scale. In contrast, the lack of geographical barriers among forest belts in the western Palaearctic during both glacial and inter-glacial epochs (see [46]), especially between central European and Mediterranean forests, may explain why forest bird communities are homogeneous over the whole western Palaearctic [46,47]. Patterns in phylogenetic diversity shown here confirm these macroecological trends.

The second version of crossed-DPCoA also highlighted, even if more marginally, the phylogenetic differences between Corsica and all other locations: avian communities within Corsica had affinities with all other locations although they can be distinguished by their higher relative abundance of Fringillidae. The relative specificity of avian communities in Corsica with a dominance of Fringillidae, may be explained by aspects of the “insular syndrome” (e.g. [48]): the fact that Corsica has phylogenetic affinities with all other locations, including non-Mediterranean species assemblages, may be explained by processes of colonisation of islands. Insular communities include only a fraction of the regional pool of species (associated with the mainland) and widespread generalist species have higher probabilities of successfully colonising islands. These effects are characteristics of the Fringillidae [49,50].

### Applications of crossed-DPCoA in ecological studies

Like several other two-way ordination techniques, crossed-DPCoA allows the effects of two factors to be distinguished where no such effects could be discerned from simpler analysis [[23,^43^]. Here we discuss some attributes and advantages of crossed-DPCoA including (i) a comparison between the two versions of the analysis; (ii) a connection with well-used statistical methodologies, leading to a consistent framework for analysing the explanatory factors that affect the composition of communities; and (iii) the broader range of application in ecology.

#### (i) Pros and cons of the two versions of the analysis

The re-centring process used in version 1 of crossed-DPCoA has been chosen in many ordination analyses to examine the effect of a factor given a co-factor (see for instance [40,51]). Version 2 also performs this re-centring process but goes one step further: in the space of DPCoA, it eliminates the entire subspace generated by the positions of the levels of the co-factor. A property of the first version is that, when the re-centring process is applied to the extended space of DPCoA, the inertia of communities’ points is *SS*(A)+*SS*(A,B) (the total effects of A, including its main effect and interaction with B) and the inertia of the positions of the levels of factor A is *SS*(A). In the second version, projecting all points from the space of DPCoA to the orthogonal complement of the space generated by the levels of the co-factor B leads to a loss of inertia: the inertia of communities’ points is lower than or equal to *SS*(A)+*SS*(A,B), and the inertia of the positions of the levels of factor A lower than or equal to *SS*(A). The two versions can thus be applied successively and their results compared. Version 2 is useful when the factor A is known a priori to present some correlation with factor B. For instance, in the case study we analysed in this study, vegetation height in Poland was taller than in any other regions throughout the successional gradient (Table 1 in [42]). This might explain the similarities found between the first axis of crossed-DPCoA version 1, that demonstrated differences between Algeria and Poland, and the first axis of the analysis of the main effect of B that highlighted the successional gradient.

For both versions, the results depend on species’ proportions within communities and on the (phylogenetic, functional) dissimilarities among species. To evaluate the relative impact of species’ proportions versus the dissimilarities among species on the results of our approach, a potential solution would be to run successively the approach considering species’ proportions and then presence/absence and considering the distances among species of interest (as done here) and then setting the distances among species equal to a constant (in that case species will be said to be equidistant). This solution compares traditional analyses of biodiversity which considered species as equidistant with new approaches that integrate species’ characteristics such as functional traits or phylogeny (e.g. [2]). It also compares biodiversity patterns based on presence/absence with those based on species’ proportions (e.g. relative abundance, biomass or density). Many studies of phylogenetic diversity have dealt with presence?absence data, which are likely to miss important ecological patterns [52]. Previous studies confirmed that the strength of phylogenetic signal in communities can be changed by considering presence ? absence data vs. proportion data (e.g. [53]).

#### (ii) Crossed-DPCoA can include various ways of comparing ecological communities

We applied crossed-DPCoA to the analysis of phylogenetic diversity in avian communities along successional forest gradients. Obviously, our approach could be applied to a wide range of ecological questions. First as highlighted in the section *Materials and Methods*, the values used to define species’ proportions can take different forms (e.g. in terms of biomass, abundance, density, and presence-absence). The approach may also be used to analyse any type of biodiversity (values in **Δ**, e.g. based on taxonomic, phylogenetic, or functional data).

Crossed-DPCoA generalizes the simple DPCoA approach which itself is a generalization of several approaches widely used in ecology but the applications of which are more limited in the context of biodiversity analyses [17]. We concentrate below on two of these approaches which can integrate any type of species’ proportions as shown in Text S7: non-symmetrical correspondence analysis [23,24] and canonical correspondence analysis [21,22].

When species are equidistant, DPCoA is equal to non-symmetrical correspondence analysis ([23,24], proof in [17] and Text S7). Applied to the table with species as rows, communities as columns and some value of species’ relative importance as entry (such as abundances, biomass, densities, or simply in terms of presence-absence), the objective of non-symmetrical correspondence analysis is to evaluate whether the relative importance of a species depends on the community in which it occurs. In this particular case, species are considered to be equidistant. The crossed-DPCoA can also be applied to equidistances among species (proof in Text S7). It provides an alternative to previous analyses developed to evaluate the relative effects of two crossed factors on the compositions of communities in terms of the chosen value of species’ importance but without considering functional or phylogenetic distances among species. These previous analyses are partial non-symmetrical correspondence analysis [23], partial canonical correspondence analysis [54] and Foucart’s analysis [43]. Crossed-DPCoA applied to species equidistance, partial non-symmetrical correspondence analysis, partial canonical correspondence analysis and Foucart’s analysis differ in the object of interest (i.e. factor A, B, and/or interactions) and in the way this object is analysed as shown in Text S7: crossed-DPCoA applied to species equidistance and partial canonical correspondence analysis both analyse the main effect of A and the interaction between A and B independently of the main effect of B but partial canonical correspondence analysis relies on correspondence analysis (where species and communities have symmetrical roles) whereas crossed-DPCoA relies on asymmetrical correspondence analysis (where differences among communities are analysed thanks to the species they contain, i.e. communities constitute the target of the study); partial non-symmetrical correspondence analysis compares species’ proportions within communities with their average proportions over all levels of factor A but per level of factor B; finally Foucart’s analysis displays first the main effect of A averaged over all levels of factor B and then reveals how this averaged effect is affected by interactions between A and B.

As many analyses developed in ecology, canonical correspondence analysis (CCA, [21,22]) is used almost exclusively in the context in which it has been developed: the analysis of species’ niches. Here a first table contains non-negative integers, usually abundances of species in sites, and a second table contains environmental variables that characterize the sites. However CCA is actually flexible in the data it can handle. In particular it can be applied to a first table that contains abundances of species in sites and a second table that contains functional traits of species. In that context, it can be demonstrated that the DPCoA is equivalent to CCA when functional distances among species have been calculated by applying the Mahalanobis distance to the table of functional traits, as demonstrated in [17] and Text S7 (the connection between canonical correspondence analysis and Mahalanobis distance has been demonstrated and acknowledged in [55,56] and is discussed in Text S7). The Mahalanobis distance has the advantage of taking the correlations between the biological traits into account when computing distances among species. Used with species’ abundances, CCA imposes that communities are weighted by the sum of the abundances of their species. However, Text S7 provides a more general link between CCA and DPCoA where abundances might be replaced with species’ proportions defined by relative abundance, biomass or density for example. The crossed-DPCoA then provides a generalization of canonical correspondence analysis. In this context, crossed-DPCoA allows an evaluation of the effects of two interacting factors on the functional composition of communities.

One advantage of crossed-DPCoA over all these specific approaches is that it can consider different aspects of the diversity of communities. Indeed, it allows a large flexibility in the data (e.g. species identity exclusively leading to equidistances among species, biological traits, or phylogeny) and in the mathematical expressions used to compute the dissimilarities among species based on these data (any expression adapted to the data may be used e.g. Euclidean, Mahalanobis or mixed-variables coefficient of distance [29] for biological traits, sum of branch lengths for phylogenies).

The applications that can be performed using crossed-factor approaches in ecology are numerous and crossed-DPCoA provides a flexible framework to improve the inferences from these applications. Biological conservation studies might be interested for instance in the analyses of interacting factors that render communities vulnerable to such issues as climate change or alien species on native communities [57]. Another promising field of application is the analysis of interacting factors (e.g. interacting environmental gradients) in experimental studies that determine which species or traits better explain ecosystem processes (e.g. plant productivity, [58]) distinguishing between niche complementarity (communities with many different trait values) or competitive exclusion (communities with similar traits) along gradients.

#### (iii) Broader applications of the crossed-DPCoA approach

**Figure 5:**
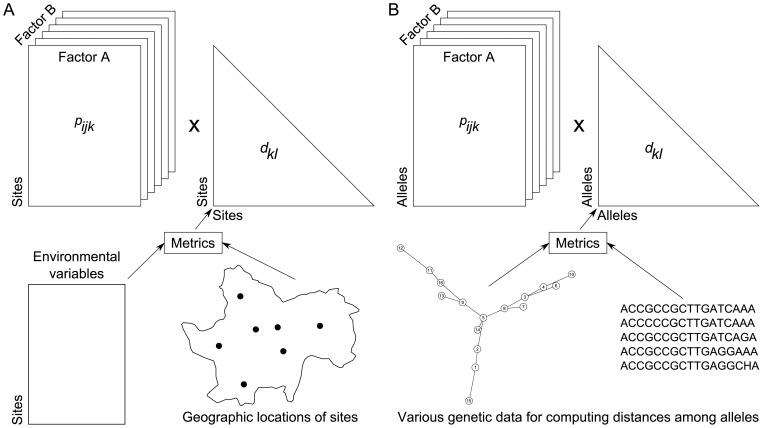
Examples of other types of data sets that might be processed using crossed-DPCoA (see Fig. 1). (A) Here the 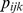 might represent the relative abundance of species *i* within site *k* measured in a particular condition *j* (say for instance year *j*). The two crossed factors in that case are species and years. The objective might be to analyse temporal changes in the similarities of environmental niches among species (change in the patterns of co-occurrence across years). Distance metrics are used to transform raw data (here tables of environmental variables) into a symmetrical matrix of distances among sites (the metrics used with species functional traits can also be used with site environmental variables, see *QE–Quadratic entropy* for details). (B) Here the 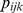 might represent the relative abundance of allele *k* within population *ij* characterized by the *i*th level of a factor A and the *j*th level of a factor B. Distance metrics are used to transform raw data (here tables that describe the alleles) into a symmetrical matrix of distances among alleles (see for instance [62]).

In this paper we have applied crossed-DPCoA to compare communities. But our approach can be used to elucidate other ecological problems (Fig. 5). In ecology, species compositions of communities in different environments are often compared. A related question is the comparison of environmental locations where different species live: analysis of species-specific environmental niches. Species can be grouped first into clades or taxonomic levels (a first factor) and then according to a categorical trait (second interacting factor) to evaluate the relative effects of species traits and phylogeny on species environmental niches [59]. If the focus is on populations of a single species, instead of communities of several species, then crossed-DPCoA can be used to analyse the genetic structure of populations and metapopulations. Individuals of several populations can then be compared based on genetic distances, such as nucleotide differences between haplotypes (e.g. [60]). For instance, it can be used to compare genetic differences among populations of nitrogen fixing bacteria influenced by geographical isolation (first factor) and host specialization (second crossed factor) [61].

There is an urgent and increasing need for methods analysing biodiversity that can integrate many explanatory factors. Critically, our methodological advances help understand those processes that might explain shifts in biodiversity (in terms of genes, taxonomy, phylogeny, or functional traits). Many different indices of biodiversity have been developed over the last 40 years. However, what is urgently required are frameworks that allow inferences to progress from answering the question of "how much biodiversity?" towards answering the question of "how does biodiversity change with potentially important factors such as biogeography, ecological processes, or anthropogenic impacts?" Crossed-DPCoA provides a useful tool for visualising and characterizing the effects of such factors and their interactions on biodiversity in factorial designs. It is essential that the full range of potential applications of this new suite of methods, for biology, ecology and genetics, be actively explored to achieve new insights into both the patterns and underlying processes governing biodiversity.

## Supporting Information

Dataset S1 **The data set in ascii format to be loaded by the R software.** The data are described in Text S4.
(RDA)

Text S1 **Notations and proofs.**
(PDF)

Text S2 **Detailed description of crossed-DPCoA, discussion and further propositions.** We provide all equations necessary to obtain the space of DPCoA and to perform crossed-DPCoA. Our choices are justified and compared with other possible versions of crossed-DPCoA. The issues related to repetition and unbalanced schemes are discussed and solutions given.
(PDF)

Text S3 **R scripts.** R scripts are used in Text S4.
(TXT)

Text S4 **Manual for R scripts.** This appendix uses data available in Dataset S1 and R scripts available in Text S3.
(PDF)

Text S5 **Species whose positions in the phylogeny were not defined by Davis.** This appendix contains details on the establishment of the phylogeny.
(PDF)

Text S6 **Bird taxonomy.**
(PDF)

Text S7 **Connections between crossed-DPCoA and other ordination approaches.** Previously developed crossed analyses that treat species as equidistant, as with classical diversity indices are compared with crossed-DPCoA.
(PDF)
