# Supplementary material for: A New Technique for Analysing Interacting Factors Affecting Biodiversity Patterns: Crossed-DPCoA
Source: PLoS One. 2013 Jan 24;8(1):e54530. doi: 10.1371/journal.pone.0054530 (PMC3554745; doi:10.1371/journal.pone.0054530)
Supplement: Text S1 — Notations and proofs. (PDF) [file pone.0054530.s002.pdf]

Text S1 – Notations and proofs

Similarly as a value of importance (proportion) is attributed to a species within a community, the communities themselves can be given different importance values in the analysis: w_{ij} will denote the importance given to community *ij*. Alternatively, similarly as species' importance can be set equal within a community (e.g. presence-absence data), communities can be attributed equal weights: $w_{ij} = 1/rm$ for all *ij*. In the main text, we have chosen equal weights for all communities. Nevertheless to demonstrate the flexibility of our approach, it is important to acknowledge that unequal weights are allowed when appropriate for a given study. In addition, as all demonstrations below are still valid with unequal weights, we present them in this more general case. Mathematically, the only requirements that cannot be violated are that $w_{ij} \geq 0$ for all *ij* and $\sum_{ij} w_{ij} = 1$. We will see, in proof 4 below and in Text S2, that an additional interesting requirement is that $w_{ij} = w_{i+}w_{+j}$ where w_{i+} is the importance given to level *i* of factor A and w_{+j} is the importance given to level *j* of factor B. Communities' importance can thus derive from the importance given to levels of a factor. Alternatively, communities' importance can be defined first and then the importance given to levels of factor A and factor B are defined as follows: $w_{i+} = \sum_{j=1}^{m} w_{ij}$ and $w_{+j} = \sum_{i=1}^{r} w_{ij}$.
Ecologically speaking, differential importance can be attributed to communities for instance when local community sizes are unequal or when communities have been sampled differentially (in that case the importance given to a community can be its relative sample size), when communities represent different areas (in which case the importance given to a community can be its relative area) (e.g., [1]). Similarly differential importance can be given to levels of a factor when the number of individuals attributed to each level is biased towards a few levels or when levels represent regions of different areas.

NOTATIONS

● **General matrices:**

$\mathbf{I}_n$ denotes the $n \times n$ matrix of identity, $\mathbf{1}_n$ denotes a $n \times 1$ vector of units, and $\mathbf{0}_n$ denotes a $n \times 1$ vector of zeros.

● **Distance matrices:**

$\boldsymbol{\Delta}$ is the matrix of distances among species.

● **Vector of weights**:

All vectors belong to

$P^S = \left\{ \mathbf{p}^t = \left(p_1, ..., p_S \right) \mid p_k \geq 0 \text{ for all } k, \sum_{k=1}^{S} p_k = 1 \right\}$, where t is the transpose.

w_{ij} is the weight attributed to community ij associated with level i of factor A and level j of factor B.

$w_{i+} = \sum_{j=1}^{m} w_{ij}$ is the weight attributed to level i of factor A.

$w_{+j} = \sum_{i=1}^{r} w_{ij}$ is the weight attributed to level j of factor B.

$p_{++k} = \sum_{i=1}^{r} \sum_{j=1}^{m} w_{ij} p_{ijk}$ is the weight attributed to species k, where p_{ijk} is the proportion of species k in community ij.

$\mathbf{w}_{\mathrm{A}}^t = \left(w_{1+}, ..., w_{i+}, ..., w_{r+} \right)$ and $\mathbf{w}_{\mathrm{B}}^t = \left(w_{+1}, ..., w_{+j}, ..., w_{+m} \right)$ are vectors of weights associated with factors A and B.

$\mathbf{w}_{\mathrm{C}}^t = \left(w_{11}, w_{12}, ..., w_{ij}, ..., w_{rm} \right)$ is the vector of weights associated with the communities.

● **Vectors and matrices of proportions**:

$\mathbf{p}_{ij}^t = \left(p_{ij1}, ..., p_{ijk}, ..., p_{ijS} \right)$ is the vector of species proportions in community ij.

$\mathbf{p}_{i+} = \sum_{j=1}^{m} w_{ij} \mathbf{p}_{ij} / w_{i+}$ is the vector of species proportions associated with level i of factor A.

$\mathbf{p}_{+j} = \sum_{i=1}^{r} w_{ij} \mathbf{p}_{ij} / w_{+j}$ is the vector of species proportions associated with level j of factor B.

$\mathbf{p}_{++} = \sum_{i=1}^{r} \sum_{j=1}^{m} w_{ij} \mathbf{p}_{ij}$ is the vector of species proportions over the whole data set.

The $S \times r$ matrix $\mathbf{P}_{\mathrm{A}} = \left[\mathbf{p}_{1+} \mathbf{p}_{2+} ... \mathbf{p}_{r+} \right]$ has species as rows and levels of factor A as columns.

The $S \times m$ matrix $\mathbf{P}_{\mathrm{B}} = \left[\mathbf{p}_{+1} \mathbf{p}_{+2} ... \mathbf{p}_{+m} \right]$ has species as rows and levels of factor B as columns.

The $S \times rm$ matrix $\mathbf{P}_{\mathrm{C}} = \left[\mathbf{p}_{11} \mathbf{p}_{12} ... \mathbf{p}_{rm} \right]$ has species as rows and communities as columns.

● **Diagonal weight matrices:**

Let $\mathbf{W}_S = diag(\mathbf{p}_{++})$ be the diagonal matrix with the species weights, $\mathbf{W}_{\mathrm{A}} = diag(\mathbf{w}_{\mathrm{A}})$, $\mathbf{W}_{\mathrm{B}} = diag(\mathbf{w}_{\mathrm{B}})$ and $\mathbf{W}_{\mathrm{C}} = diag(\mathbf{w}_{\mathrm{C}})$.

● **Centring matrix**:

$\mathbf{Q} = \mathbf{I}_S - \mathbf{1}_S \mathbf{1}_S^t \mathbf{W}_S$

THE SPACE OF DPCOA

With the notations given above, the weighted principal coordinated analysis (PCoA) of the Euclidean matrix $\mathbf{\Delta}$=(δ_{kl}) defines a space where species, communities and levels of factors are positioned. It consists in

$-\mathbf{QDQ} = \mathbf{XX}^t$

where the $S \times v$ matrix $\mathbf{X}$ gives the coordinates (per row) of each species and $\mathbf{D} = \left(\delta_{kl}^2/2\right)$. v is the number of axes of this space (see [2]). As specified in the main text, the coordinates of the communities, levels of factor A and levels of factor B are given by matrices $\mathbf{Y}_{\mathrm{A}} = \mathbf{P}_{\mathrm{A}}^t \mathbf{X}$; $\mathbf{Y}_{\mathrm{B}} = \mathbf{P}_{\mathrm{B}}^t \mathbf{X}$; $\mathbf{Y}_{\mathrm{C}} = \mathbf{P}_{\mathrm{C}}^t \mathbf{X}$, respectively.

PROOF 1 – PROOF THAT THE MEASURES OF POINT DISPERSIONS, IN THE SPACE OF DPCOA, CORRESPOND TO THE CROSSED DECOMPOSITION OF QUADRATIC ENTROPY

For the general notations, see above.

Given we restricted discussion to the situations where $\mathbf{\Delta}$=(δ_{kl}) is Euclidean, there exists a Euclidean space with S points M_k, so that $\|M_k M_l\| = \delta_{kl}$ for all k and l. This space can be obtained through a Principal Coordinate Analysis (PCoA).

As highlighted above, the weighted PCoA of matrix $\mathbf{\Delta}$=(δ_{kl}) consists in

$-\mathbf{QDQ} = \mathbf{XX}^t$

where the $S \times v$ matrix $\mathbf{X}$ gives the coordinates (per row) of each species and $\mathbf{D} = \left(\delta_{kl}^2/2\right)$.

The coordinates of the communities, levels of factor A and levels of factor B are given by matrices $\mathbf{Y}_{\mathrm{A}} = \mathbf{P}_{\mathrm{A}}^t \mathbf{X}$; $\mathbf{Y}_{\mathrm{B}} = \mathbf{P}_{\mathrm{B}}^t \mathbf{X}$; $\mathbf{Y}_{\mathrm{C}} = \mathbf{P}_{\mathrm{C}}^t \mathbf{X}$, respectively.

Let M_k, C_{ij}, A_i, B_j and G be the points corresponding to species k, community ij, level i of factor A, level j of factor B and the centre of the Euclidean space, respectively. Let $\mathbf{x}_k$ be the vector that correspond to the kth row of $\mathbf{X}$ and contains the coordinates of M_k. The vectors $\mathbf{c}_{ij}$, $\mathbf{a}_i$ and $\mathbf{b}_j$ are the ijth row of $\mathbf{Y}_\text{C}$, the ith row of $\mathbf{Y}_\text{A}$ and the jth row of $\mathbf{Y}_\text{B}$, respectively. These vectors contain the coordinates of the points C_{ij}, A_i, and B_j, respectively. Denote $\mathbf{g}$ the vector of 0 corresponding to the coordinates of G. These vectors verify the following relationships:

$$\mathbf{c}_{ij} = \mathbf{X}^t\mathbf{p}_{ij} = \sum_{k=1}^{S} p_{ijk}\mathbf{x}_k ,$$

and $$\sum_{i=1}^{r}\sum_{j=1}^{m} w_{ij}\mathbf{c}_{ij} = \sum_{i=1}^{r}\sum_{j=1}^{m}\sum_{k=1}^{S} w_{ij}p_{ijk}\mathbf{x}_k = \sum_{k=1}^{S}\left(\sum_{i=1}^{r}\sum_{j=1}^{m} w_{ij}p_{ijk}\right)\mathbf{x}_k = \sum_{k=1}^{S} p_{++k}\mathbf{x}_k$$

$$\mathbf{a}_i = \mathbf{X}^t\mathbf{p}_{i+} = \sum_{k=1}^{S}\sum_{j=1}^{m}\frac{w_{ij}}{w_{i+}} p_{ijk}\mathbf{x}_k ,$$

and $$\sum_{i=1}^{r} w_{i+}\mathbf{a}_i = \sum_{i=1}^{r} w_{i+}\sum_{k=1}^{S}\sum_{j=1}^{m}\frac{w_{ij}}{w_{i+}} p_{ijk}\mathbf{x}_k = \sum_{k=1}^{S}\left(\sum_{i=1}^{r}\sum_{j=1}^{m} w_{ij}p_{ijk}\right)\mathbf{x}_k = \sum_{k=1}^{S} p_{++k}\mathbf{x}_k$$

$$\mathbf{b}_j = \mathbf{X}^t\mathbf{p}_{+j} = \sum_{k=1}^{S}\sum_{i=1}^{r}\frac{w_{ij}}{w_{+j}} p_{ijk}\mathbf{x}_k$$

and $$\sum_{j=1}^{m} w_{+j}\mathbf{b}_j = \sum_{j=1}^{m} w_{+j}\sum_{k=1}^{S}\sum_{i=1}^{r}\frac{w_{ij}}{w_{+j}} p_{ijk}\mathbf{x}_k = \sum_{k=1}^{S}\left(\sum_{i=1}^{r}\sum_{j=1}^{m} w_{ij}p_{ijk}\right)\mathbf{x}_k = \sum_{k=1}^{S} p_{++k}\mathbf{x}_k$$

This leads to $$\sum_{i=1}^{r} w_{i+}\mathbf{a}_i = \sum_{j=1}^{m} w_{+j}\mathbf{b}_j = \sum_{i=1}^{r}\sum_{j=1}^{m} w_{ij}\mathbf{c}_{ij} = \sum_{k=1}^{S} p_{++k}\mathbf{x}_k$$

By definition of the weighted PCoA,

$$\sum_{k=1}^{S} p_{++k}\mathbf{x}_k = (0,...,0)^t = \mathbf{g} ,$$

which completes the demonstration that all sets of points (for species, communities, levels of factor A, levels of factor B) are centred for their respective weights.

We now have to demonstrate that $\frac{1}{2}\left\|A_iA_{i'}\right\|^2 = D_\Delta\left(\mathbf{p}_{i+},\mathbf{p}_{i'+}\right)$, $\frac{1}{2}\left\|B_iB_{j'}\right\|^2 = D_\Delta\left(\mathbf{p}_{+j},\mathbf{p}_{+j'}\right)$, $\frac{1}{2}\left\|C_{ij}C_{i'j'}\right\|^2 = D_\Delta\left(\mathbf{p}_{ij},\mathbf{p}_{i'j'}\right)$. It can be noted that for any vectors $\mathbf{p}$ and $\mathbf{q}$ in P^S [2]:

$$D_\Delta(\mathbf{p},\mathbf{q}) = -\tfrac{1}{2}(\mathbf{p}-\mathbf{q})^t\,\mathbf{D}(\mathbf{p}-\mathbf{q})$$

where $\mathbf{D} = \left(\delta_{kl}^2 / 2\right)$ (see above).

Given that $\mathbf{p}^t\mathbf{1}_S = 1$, $\mathbf{p}^t\mathbf{Q} = \mathbf{p}^t\left(\mathbf{I}_S - \mathbf{1}_S\mathbf{1}_S^t\mathbf{W}_S\right) = \mathbf{p}^t - \mathbf{p}^t\mathbf{1}_S\mathbf{1}_S^t\mathbf{W}_S = \mathbf{p}^t - \mathbf{1}_S^t\mathbf{W}_S$. Then,

$$\left(\mathbf{p}-\mathbf{q}\right)^t\mathbf{Q} = \left(\mathbf{p}^t - \mathbf{1}_S^t\mathbf{W}_S\right) - \left(\mathbf{q}^t - \mathbf{1}_S^t\mathbf{W}_S\right) = \left(\mathbf{p}-\mathbf{q}\right)^t$$

This leads to

$$\begin{aligned} D_\Delta\left(\mathbf{p},\mathbf{q}\right) &= \tfrac{1}{2}\left(\mathbf{p}-\mathbf{q}\right)^t\left(-\mathbf{QDQ}\right)\left(\mathbf{p}-\mathbf{q}\right) \\ &= \tfrac{1}{2}\left(\mathbf{p}-\mathbf{q}\right)^t\mathbf{XX}^t\left(\mathbf{p}-\mathbf{q}\right) \\ &= \tfrac{1}{2}\left(\mathbf{X}^t\mathbf{p}-\mathbf{X}^t\mathbf{q}\right)^t\left(\mathbf{X}^t\mathbf{p}-\mathbf{X}^t\mathbf{q}\right) \end{aligned}$$

Because, $\mathbf{X}^t\mathbf{p}_{i+} = \mathbf{a}_i$, $\mathbf{X}^t\mathbf{p}_{+j} = \mathbf{b}_j$ and $\mathbf{X}^t\mathbf{p}_{ij} = \mathbf{c}_j$, it follows that

$$D_\Delta\left(\mathbf{p}_{i+},\mathbf{p}_{i'+}\right) = \tfrac{1}{2}\left(\mathbf{a}_i - \mathbf{a}_{i'}\right)^t\left(\mathbf{a}_i - \mathbf{a}_{i'}\right)$$

$$D_\Delta\left(\mathbf{p}_{+j},\mathbf{p}_{+j'}\right) = \tfrac{1}{2}\left(\mathbf{b}_j - \mathbf{b}_{j'}\right)^t\left(\mathbf{b}_j - \mathbf{b}_{j'}\right)$$

$$D_\Delta\left(\mathbf{p}_{ij},\mathbf{p}_{i'j'}\right) = \tfrac{1}{2}\left(\mathbf{c}_{ij} - \mathbf{c}_{i'j'}\right)^t\left(\mathbf{c}_{ij} - \mathbf{c}_{i'j'}\right)$$

It can now be easily demonstrated that the components of diversity in the analysis of quadratic entropy (ANOQE) are measures of multivariate point dispersion:

$$SST = \sum_{k=1}^{S}\sum_{l=1}^{S} p_{++k}p_{++l}\frac{\delta_{kl}^2}{2} = \frac{1}{2}\sum_{k=1}^{S}\sum_{l=1}^{S} p_{++k}p_{++l}\left\|M_kM_l\right\|^2$$

$$SSA = \sum_{i=1}^{r}\sum_{i'=1}^{r} w_{i+}w_{i'+}D_\Delta\left(\mathbf{p}_{i+},\mathbf{p}_{i'+}\right) = \frac{1}{2}\sum_{i=1}^{r}\sum_{i'=1}^{r} w_{i+}w_{i'+}\left\|A_iA_{i'}\right\|^2$$

$$SSB = \sum_{j=1}^{m}\sum_{j'=1}^{m} w_{+j}w_{+j'}D_\Delta\left(\mathbf{p}_{+j},\mathbf{p}_{+j'}\right) = \frac{1}{2}\sum_{j=1}^{m}\sum_{j'=1}^{m} w_{+j}w_{+j'}\left\|B_jB_{j'}\right\|^2$$

$$SSW = \frac{1}{2}\sum_{i=1}^{r}\sum_{j=1}^{m} w_{ij}\sum_{k=1}^{S}\sum_{l=1}^{S} p_{ijk}p_{ijl}\delta_{kl}^2 = \frac{1}{2}\sum_{i=1}^{r}\sum_{j=1}^{m} w_{ij}\sum_{k=1}^{S}\sum_{l=1}^{S} p_{ijk}p_{ijl}\left\|M_kM_l\right\|^2$$

In addition simple developments give that (see for instance [2]),

$$SST = \sum_{k=1}^{S} p_{++k}\left\|M_kG\right\|^2$$

$$SSA = \sum_{i=1}^{r} w_{i+}\left\|A_iG\right\|^2$$

$$SSB = \sum_{j=1}^{m} w_{+j}\left\|B_jG\right\|^2$$

$$SSW = \sum_{i=1}^{r} \sum_{j=1}^{m} w_{ij} \sum_{k=1}^{S} p_{ijk} \left\| M_k C_{ij} \right\|^2$$

See proof 4 below for the component of interaction.

PROOF 2 – CROSSED-DPCOA VERSION 2: PROOF THAT THE ORTHOGONAL PROJECTOR IN THE SPACE OF THE LEVELS OF FACTOR B IS $\mathbf{\Pi}_{\mathrm{B}} = \mathbf{Y}_{\mathrm{B}}^t \left(\mathbf{Y}_{\mathrm{B}} \mathbf{Y}_{\mathrm{B}}^t \right)^{-1} \mathbf{Y}_{\mathrm{B}}$ (USED IN TEXT S2).

The principal axes and principal components of the points corresponding to the levels of B are given by the generalized singular value decomposition of the triplet $\left(\mathbf{Y}_{\mathrm{B}}, \mathbf{I}_{\nu}, \mathbf{W}_{\mathrm{B}} \right)$:

$$\mathbf{Y}_{\mathrm{B}} = \mathbf{V} \mathbf{\Lambda}^{1/2} \mathbf{U}^t$$

$$\mathbf{Y}_{\mathrm{B}}^t \mathbf{W}_{\mathrm{B}} \mathbf{Y}_{\mathrm{B}} = \mathbf{U} \mathbf{\Lambda} \mathbf{U}^t$$

$$\mathbf{Y}_{\mathrm{B}} \mathbf{Y}_{\mathrm{B}}^t = \mathbf{V} \mathbf{\Lambda} \mathbf{V}^t$$

The matrices $\mathbf{U}$ and $\mathbf{V}$ verify $\mathbf{U}^t \mathbf{U} = \mathbf{I}_m$ and $\mathbf{V}^t \mathbf{W}_{\mathrm{B}} \mathbf{V} = \mathbf{I}_{\nu}$. The orthogonal projector in the space of B is $\mathbf{U} \left(\mathbf{U}^t \mathbf{U} \right)^{-1} \mathbf{U}^t = \mathbf{U} \mathbf{U}^t$. The following relationships link the principal axes with the principal components: $\mathbf{V} = \mathbf{Y}_{\mathrm{B}} \mathbf{U} \mathbf{\Lambda}^{-1/2}$ and $\mathbf{U} = \mathbf{Y}_{\mathrm{B}}^t \mathbf{W}_{\mathrm{B}} \mathbf{V} \mathbf{\Lambda}^{-1/2}$. Thus

$\mathbf{U} \mathbf{U}^t = \mathbf{Y}_{\mathrm{B}}^t \mathbf{W}_{\mathrm{B}} \mathbf{V} \mathbf{\Lambda}^{-1} \mathbf{V}^t \mathbf{W}_{\mathrm{B}} \mathbf{Y}_{\mathrm{B}}$. Given that

$$\mathbf{V} \mathbf{\Lambda} \mathbf{V}^t \left(\mathbf{W}_{\mathrm{B}} \mathbf{V} \mathbf{\Lambda}^{-1} \mathbf{V}^t \mathbf{W}_{\mathrm{B}} \right) \mathbf{V} \mathbf{\Lambda} \mathbf{V}^t = \mathbf{V} \mathbf{\Lambda} \mathbf{V}^t,$$

then

$$\mathbf{W}_{\mathrm{B}} \mathbf{V} \mathbf{\Lambda}^{-1} \mathbf{V}^t \mathbf{W}_{\mathrm{B}} = \left(\mathbf{V} \mathbf{\Lambda} \mathbf{V}^t \right)^{-1} = \left(\mathbf{Y}_{\mathrm{B}} \mathbf{Y}_{\mathrm{B}}^t \right)^{-1}.$$

This leads to $\mathbf{U} \mathbf{U}^t = \mathbf{Y}_{\mathrm{B}}^t \left(\mathbf{Y}_{\mathrm{B}} \mathbf{Y}_{\mathrm{B}}^t \right)^{-1} \mathbf{Y}_{\mathrm{B}}$.

PROOF 3 – CROSSED-DPCOA VERSION 2: PROOF THAT THE FINAL COORDINATES ARE CENTRED

The demonstration is immediate by the fact that intermediate coordinates $\mathbf{X}$, $\mathbf{Y}_{\mathrm{A}}$, $\mathbf{Y}_{\mathrm{B}}$ and $\mathbf{Y}_{\mathrm{C}}$ are centred (*i.e.* $\mathbf{X}^t \mathbf{W}_S \mathbf{1}_S = \mathbf{0}_S$, $\mathbf{Y}_{\mathrm{A}}^t \mathbf{W}_{\mathrm{A}} \mathbf{1}_r = \mathbf{0}_r$, $\mathbf{Y}_{\mathrm{B}}^t \mathbf{W}_{\mathrm{B}} \mathbf{1}_m = \mathbf{0}_m$, $\mathbf{Y}_{\mathrm{C}}^t \mathbf{W}_{\mathrm{C}} \mathbf{1}_{rm} = \mathbf{0}_{rm}$):

PROOF 4 – SIMPLE EXPRESSION FOR THE COMPONENT OF INTERACTION

A simple expression for the component of interaction can be obtained when $w_{ij}=w_{i+}w_{+j}$. Let Σ_{ij} be a point located at coordinates $(\mathbf{p}_{ij}\text{-}\mathbf{p}_{i+}\text{-}\mathbf{p}_{+j}+\mathbf{p}_{++})^t\mathbf{X}$. This point represents a position community ij would have if all positions of the levels of factor A and those of the levels of factor B were moved to the centre of the space of DPCoA. This re-centring process would remove the main effects of A and B. With these notations, the inertia of points Σ_{ij} for all i and j would be

$$SS(A,B)=\sum_{i=1}^{r}\sum_{j=1}^{m}\sum_{i'=1}^{r}\sum_{j'=1}^{m}w_{ij}w_{i'j'}\frac{\left\|\Sigma_{ij}\Sigma_{i'j'}\right\|^2}{2}$$

Proof:

$$\left\|\Sigma_{ij}\Sigma_{i'j'}\right\|=\left(\mathbf{p}_{ij}-\mathbf{p}_{i+}-\mathbf{p}_{+j}+\mathbf{p}_{++}-\mathbf{p}_{i'j'}+\mathbf{p}_{i'+}+\mathbf{p}_{+j'}-\mathbf{p}_{++}\right)^t\mathbf{XX}^t\left(\mathbf{p}_{ij}-\mathbf{p}_{i+}-\mathbf{p}_{+j}+\mathbf{p}_{++}-\mathbf{p}_{i'j'}+\mathbf{p}_{i'+}+\mathbf{p}_{+j'}-\mathbf{p}_{++}\right)$$

$$\left\|\Sigma_{ij}\Sigma_{i'j'}\right\|=\left(\left[\mathbf{p}_{ij}-\mathbf{p}_{i'j'}\right]-\left[\mathbf{p}_{i+}-\mathbf{p}_{i'+}\right]-\left[\mathbf{p}_{+j}-\mathbf{p}_{+j'}\right]\right)^t\mathbf{XX}^t\left(\left[\mathbf{p}_{ij}-\mathbf{p}_{i'j'}\right]-\left[\mathbf{p}_{i+}-\mathbf{p}_{i'+}\right]-\left[\mathbf{p}_{+j}-\mathbf{p}_{+j'}\right]\right)$$

$$\begin{aligned}\left\|\Sigma_{ij}\Sigma_{i'j'}\right\|=&\left[\mathbf{p}_{ij}-\mathbf{p}_{i'j'}\right]^t\mathbf{XX}^t\left[\mathbf{p}_{ij}-\mathbf{p}_{i'j'}\right]+\left[\mathbf{p}_{i+}-\mathbf{p}_{i'+}\right]^t\mathbf{XX}^t\left[\mathbf{p}_{i+}-\mathbf{p}_{i'+}\right]+\left[\mathbf{p}_{+j}-\mathbf{p}_{+j'}\right]^t\mathbf{XX}^t\left[\mathbf{p}_{+j}-\mathbf{p}_{+j'}\right]\\&-\left[\mathbf{p}_{ij}-\mathbf{p}_{i'j'}\right]^t\mathbf{XX}^t\left[\mathbf{p}_{i+}-\mathbf{p}_{i'+}\right]-\left[\mathbf{p}_{i+}-\mathbf{p}_{i'+}\right]^t\mathbf{XX}^t\left[\mathbf{p}_{ij}-\mathbf{p}_{i'j'}\right]\\&-\left[\mathbf{p}_{ij}-\mathbf{p}_{i'j'}\right]^t\mathbf{XX}^t\left[\mathbf{p}_{+j}-\mathbf{p}_{+j'}\right]-\left[\mathbf{p}_{+j}-\mathbf{p}_{+j'}\right]^t\mathbf{XX}^t\left[\mathbf{p}_{ij}-\mathbf{p}_{i'j'}\right]\\&+\left[\mathbf{p}_{i+}-\mathbf{p}_{i'+}\right]^t\mathbf{XX}^t\left[\mathbf{p}_{+j}-\mathbf{p}_{+j'}\right]+\left[\mathbf{p}_{+j}-\mathbf{p}_{+j'}\right]^t\mathbf{XX}^t\left[\mathbf{p}_{i+}-\mathbf{p}_{i'+}\right]\end{aligned}$$

$$\begin{aligned}\left\|\Sigma_{ij}\Sigma_{i'j'}\right\|=&\left[\mathbf{p}_{ij}-\mathbf{p}_{i'j'}\right]^t\mathbf{XX}^t\left[\mathbf{p}_{ij}-\mathbf{p}_{i'j'}\right]+\left[\mathbf{p}_{i+}-\mathbf{p}_{i'+}\right]^t\mathbf{XX}^t\left[\mathbf{p}_{i+}-\mathbf{p}_{i'+}\right]+\left[\mathbf{p}_{+j}-\mathbf{p}_{+j'}\right]^t\mathbf{XX}^t\left[\mathbf{p}_{+j}-\mathbf{p}_{+j'}\right]\\&-2\left[\mathbf{p}_{ij}-\mathbf{p}_{i'j'}\right]^t\mathbf{XX}^t\left[\mathbf{p}_{i+}-\mathbf{p}_{i'+}\right]\\&-2\left[\mathbf{p}_{ij}-\mathbf{p}_{i'j'}\right]^t\mathbf{XX}^t\left[\mathbf{p}_{+j}-\mathbf{p}_{+j'}\right]\\&+2\left[\mathbf{p}_{i+}-\mathbf{p}_{i'+}\right]^t\mathbf{XX}^t\left[\mathbf{p}_{+j}-\mathbf{p}_{+j'}\right]\end{aligned}$$

$$\sum_{i=1}^{r}\sum_{j=1}^{m}\sum_{i'=1}^{r}\sum_{j'=1}^{m} w_{ij}w_{i'j'}\frac{\left\|\Sigma_{ij}\Sigma_{i'j'}\right\|^2}{2} = \frac{1}{2}\sum_{i=1}^{r}\sum_{j=1}^{m}\sum_{i'=1}^{r}\sum_{j'=1}^{m} w_{ij}w_{i'j'}\left[\mathbf{p}_{ij}-\mathbf{p}_{i'j'}\right]^t \mathbf{XX}^t\left[\mathbf{p}_{ij}-\mathbf{p}_{i'j'}\right]$$

$$+\frac{1}{2}\sum_{i=1}^{r}\sum_{j=1}^{m}\sum_{i'=1}^{r}\sum_{j'=1}^{m} w_{ij}w_{i'j'}\left[\mathbf{p}_{i+}-\mathbf{p}_{i'+}\right]^t \mathbf{XX}^t\left[\mathbf{p}_{i+}-\mathbf{p}_{i'+}\right]$$

$$+\frac{1}{2}\sum_{i=1}^{r}\sum_{j=1}^{m}\sum_{i'=1}^{r}\sum_{j'=1}^{m} w_{ij}w_{i'j'}\left[\mathbf{p}_{+j}-\mathbf{p}_{+j'}\right]^t \mathbf{XX}^t\left[\mathbf{p}_{+j}-\mathbf{p}_{+j'}\right]$$

$$-\sum_{i=1}^{r}\sum_{j=1}^{m}\sum_{i'=1}^{r}\sum_{j'=1}^{m} w_{ij}w_{i'j'}\left[\mathbf{p}_{ij}-\mathbf{p}_{i'j'}\right]^t \mathbf{XX}^t\left[\mathbf{p}_{i+}-\mathbf{p}_{i'+}\right]$$

$$-\sum_{i=1}^{r}\sum_{j=1}^{m}\sum_{i'=1}^{r}\sum_{j'=1}^{m} w_{ij}w_{i'j'}\left[\mathbf{p}_{ij}-\mathbf{p}_{i'j'}\right]^t \mathbf{XX}^t\left[\mathbf{p}_{+j}-\mathbf{p}_{+j'}\right]$$

$$+\sum_{i=1}^{r}\sum_{j=1}^{m}\sum_{i'=1}^{r}\sum_{j'=1}^{m} w_{ij}w_{i'j'}\left[\mathbf{p}_{i+}-\mathbf{p}_{i'+}\right]^t \mathbf{XX}^t\left[\mathbf{p}_{+j}-\mathbf{p}_{+j'}\right]$$

Because $w_{ij}=w_{i+}w_{+j}$,

$$\sum_{i=1}^{r}\sum_{j=1}^{m}\sum_{i'=1}^{r}\sum_{j'=1}^{m} w_{ij}w_{i'j'}\frac{\left\|\Sigma_{ij}\Sigma_{i'j'}\right\|^2}{2} = \frac{1}{2}\sum_{i=1}^{r}\sum_{j=1}^{m}\sum_{i'=1}^{r}\sum_{j'=1}^{m} w_{ij}w_{i'j'}\left\|C_{ij}C_{i'j'}\right\|^2$$

$$+\frac{1}{2}\sum_{i=1}^{r}\sum_{i'=1}^{r} w_{i+}w_{i'+}\left\|A_{i+}A_{i'+}\right\|^2$$

$$+\frac{1}{2}\sum_{j=1}^{m}\sum_{j'=1}^{m} w_{+j}w_{+j'}\left\|B_{+j}B_{+j'}\right\|^2$$

$$-\sum_{i=1}^{r}\sum_{i'=1}^{r} w_{i+}w_{i'+}\left\|A_{i+}A_{i'+}\right\|^2$$

$$-\sum_{j=1}^{m}\sum_{j'=1}^{m} w_{+j}w_{+j'}\left\|B_{+j}B_{+j'}\right\|^2$$

$$+\left[\mathbf{p}_{++}-\mathbf{p}_{++}\right]^t \mathbf{XX}^t\left[\mathbf{p}_{++}-\mathbf{p}_{++}\right]$$

$$\sum_{i=1}^{r}\sum_{j=1}^{m}\sum_{i'=1}^{r}\sum_{j'=1}^{m} w_{ij}w_{i'j'}\frac{\left\|\Sigma_{ij}\Sigma_{i'j'}\right\|^2}{2} = \frac{1}{2}\sum_{i=1}^{r}\sum_{j=1}^{m}\sum_{i'=1}^{r}\sum_{j'=1}^{m} w_{ij}w_{i'j'}\left\|C_{ij}C_{i'j'}\right\|^2$$

$$-\frac{1}{2}\sum_{i=1}^{r}\sum_{i'=1}^{r} w_{i+}w_{i'+}\left\|A_{i+}A_{i'+}\right\|^2$$

$$-\frac{1}{2}\sum_{j=1}^{m}\sum_{j'=1}^{m} w_{+j}w_{+j'}\left\|B_{+j}B_{+j'}\right\|^2$$

$$\sum_{i=1}^{r}\sum_{j=1}^{m}\sum_{i'=1}^{r}\sum_{j'=1}^{m} w_{ij}w_{i'j'}\frac{\left\|\Sigma_{ij}\Sigma_{i'j'}\right\|^2}{2} = SS(C)-SS(A)-SS(B)$$

References

1. Lande R (1996) Statistics and partitioning of species diversity, and similarity among multiple communities. Oikos 76: 5-13.
2. Pavoine S, Dufour AB, Chessel D (2004) From dissimilarities among species to dissimilarities among communities: a double principal coordinate analysis. J Theor Biol 228: 523-537.
