# Supplementary material for: A New Technique for Analysing Interacting Factors Affecting Biodiversity Patterns: Crossed-DPCoA
Source: PLoS One. 2013 Jan 24;8(1):e54530. doi: 10.1371/journal.pone.0054530 (PMC3554745; doi:10.1371/journal.pone.0054530)
Supplement: Text S2 — Detailed description of crossed-DPCoA, discussion and further propositions. We provide all equations necessary to obtain the space of DPCoA and to perform crossed-DPCoA. Our choices are justified and compared with other possible versions of crossed-DPCoA. The issues related to repetition and unbalanced schemes are discussed and solutions given. (PDF) [file pone.0054530.s003.pdf]

Text S2. Detailed description of crossed-DPCoA, discussion and further propositions

A summary of all notations was given in Text S1. For the appendixes to be read independently, we recall these notations below.

NOTATIONS

● **General matrices:**

$\mathbf{I}_n$ denotes the $n \times n$ matrix of identity, $\mathbf{1}_n$ denotes a $n \times 1$ vector of units, and $\mathbf{0}_n$ denotes a $n \times 1$ vector of zeros.

● **Distance matrices:**

$\mathbf{\Delta}$ is the matrix of distances among species.

● **Vector of weights**:

All vectors belong to

$P^S = \left\{ \mathbf{p}^t = (p_1, ..., p_S) \mid p_k \geq 0 \text{ for all } k, \sum_{k=1}^{S} p_k = 1 \right\}$, where t is the transpose.

w_{ij} is the weight attributed to community ij associated with level i of factor A and level j of factor B.

$w_{i+} = \sum_{j=1}^{m} w_{ij}$ is the weight attributed to level i of factor A.

$w_{+j} = \sum_{i=1}^{r} w_{ij}$ is the weight attributed to level j of factor B.

$p_{++k} = \sum_{i=1}^{r} \sum_{j=1}^{m} w_{ij} p_{ijk}$ is the weight attributed to species k, where p_{ijk} is the proportion of species k in community ij.

$\mathbf{w}_{\mathrm{A}}^t = (w_{1+}, ..., w_{i+}, ..., w_{r+})$ and $\mathbf{w}_{\mathrm{B}}^t = (w_{+1}, ..., w_{+j}, ..., w_{+m})$ are vectors of weights associated with factors A and B.

$\mathbf{w}_{\mathrm{C}}^t = (w_{11}, w_{12}, ..., w_{ij}, ..., w_{rm})$ is the vector of weights associated with the communities.

● **Vectors and matrices of proportions**:

$\mathbf{p}_{ij}^t = (p_{ij1}, ..., p_{ijk}, ..., p_{ijS})$ is the vector of species proportions in community ij.

$\mathbf{p}_{i+} = \sum_{j=1}^{m} w_{ij} \mathbf{p}_{ij} / w_{i+}$ is the vector of species proportions associated with level i of factor A.

$\mathbf{p}_{+j} = \sum_{i=1}^{r} w_{ij} \mathbf{p}_{ij} / w_{+j}$ is the vector of species proportions associated with level j of factor B.

$\mathbf{p}_{++} = \sum_{i=1}^{r} \sum_{j=1}^{m} w_{ij} \mathbf{p}_{ij}$ is the vector of species proportions over the whole data set.

The $S \times r$ matrix $\mathbf{P}_{\mathrm{A}} = [\mathbf{p}_{1+} \mathbf{p}_{2+} ... \mathbf{p}_{r+}]$ has species as rows and levels of factor A as columns.

The $S \times m$ matrix $\mathbf{P}_{\mathrm{B}} = [\mathbf{p}_{+1}\mathbf{p}_{+2}...\mathbf{p}_{+m}]$ has species as rows and levels of factor B as columns.

The $S \times rm$ matrix $\mathbf{P}_{\mathrm{C}} = [\mathbf{p}_{11}\mathbf{p}_{12}...\mathbf{p}_{rm}]$ has species as rows and communities as columns.

● **Diagonal weight matrices:**

Let $\mathbf{W}_S = diag(\mathbf{p}_{++})$ be the diagonal matrix with the species weights, $\mathbf{W}_{\mathrm{A}} = diag(\mathbf{w}_{\mathrm{A}})$,

$\mathbf{W}_{\mathrm{B}} = diag(\mathbf{w}_{\mathrm{B}})$ and $\mathbf{W}_{\mathrm{C}} = diag(\mathbf{w}_{\mathrm{C}})$.

● **Centring matrix**:

$\mathbf{Q} = \mathbf{I}_S - \mathbf{1}_S \mathbf{1}_S^t \mathbf{W}_S$

● **Matrices of coordinates in the space of DPCoA**:

X: $S \times v$ matrix of coordinates of the species
$\mathbf{Y}_{\mathrm{A}}$: $r \times v$ matrix of coordinates of the levels of factor A
$\mathbf{Y}_{\mathrm{B}}$: $m \times v$ matrix of coordinates of the levels of factor B
$\mathbf{Y}_{\mathrm{C}}$: $rm \times v$ matrix of coordinates of the levels of factor C

Rapid analysis of the main effect of B

The main effect of B can be analysed by obtaining the principal axes of the positions of the levels of factor B in the space of DPCoA (see Text S1 for a definition of this space). These positions are given by matrix $\mathbf{Y}_{\mathrm{B}}$. The principal axes are obtained by the following diagonalization process:

$\mathbf{Y}_{\mathrm{B}}^t \mathbf{W}_{\mathrm{B}} \mathbf{Y}_{\mathrm{B}} = \mathbf{U} \mathbf{\Lambda} \mathbf{U}^t$

U, the eigenvector matrix, contains the coordinates of the principal axes of the positions of the levels of factor B on the axes of the space of DPCoA. The coordinates of the levels of factor B on these new axes are $\mathbf{Y}_{\mathrm{B}}\mathbf{U}$, and the coordinates of the species **XU**.

Crossed-DPCoA version 1

Let $\mathbf{U}_{\mathrm{B}}$ be the matrix with communities as rows and levels of factor B as columns. The entry at the ith row and j^{th} column contains 1 if the community i is associated with the jth level of factor B and 0 otherwise. This matrix is the disjunctive matrix associated with factor B.

To move the positions of the levels of B to the centre of the space, communities' points are projected on the orthogonal complement of the subspace generated by $\mathbf{U}_{\mathrm{B}}$. The corresponding projector is

$\mathbf{P}_{\mathbf{U}_{\mathrm{B}}}^{\perp} = \mathbf{I}_{rm} - \mathbf{U}_{\mathrm{B}} \left(\mathbf{U}_{\mathrm{B}}^t \mathbf{W}_{\mathrm{C}} \mathbf{U}_{\mathrm{B}} \right)^{-1} \mathbf{U}_{\mathrm{B}}^t \mathbf{W}_{\mathrm{C}}$

The re-centred coordinates of the communities are thus in the rows of $\mathbf{P}_{\mathbf{U}_{\mathrm{B}}}^{\perp} \mathbf{Y}_{\mathrm{C}}$. A level of factor A is located at the centroid of the communities' points associated with this level. The re-centred position of a community ij is

$\mathbf{p}_{ij}^t \mathbf{X} - \mathbf{p}_{+j}^t \mathbf{X}$

The position of the level i of factor A is thus:

$$\sum_{j=1}^{m}\frac{w_{ij}}{w_{i+}}\left[\mathbf{p}_{ij}^{t}\mathbf{X}-\mathbf{p}_{+j}^{t}\mathbf{X}\right]=\sum_{j=1}^{m}\frac{w_{ij}}{w_{i+}}\mathbf{p}_{ij}^{t}\mathbf{X}-\sum_{j=1}^{m}\frac{w_{ij}}{w_{i+}}\mathbf{p}_{+j}^{t}\mathbf{X}$$

$$=\mathbf{p}_{i+}^{t}\mathbf{X}-\sum_{j=1}^{m}\frac{w_{ij}}{w_{i+}}\mathbf{p}_{+j}^{t}\mathbf{X}$$

If the importance values given to the levels of factor A and B are independent so that $w_{ij}=w_{i+}w_{+j}$ (which is the case in our main text as $w_{ij}=\frac{1}{r}\frac{1}{m}$, $w_{i+}=\frac{1}{r}$, $w_{+j}=\frac{1}{m}$), then

$$\sum_{j=1}^{m}\frac{w_{ij}}{w_{i+}}\mathbf{p}_{+j}^{t}\mathbf{X}=\sum_{j=1}^{m}w_{+j}\mathbf{p}_{+j}^{t}\mathbf{X}=\mathbf{p}_{++}^{t}\mathbf{X}$$

Given that the $\mathbf{p}_{++}^{t}\mathbf{X}=\mathbf{0}_{v}^{t}$, the $v\times 1$ vector of zeros, this leads to the position of the level i of factor A is $\mathbf{p}_{i+}^{t}\mathbf{X}$ (i.e. the position is unchanged).

If $w_{ij}=w_{i+}w_{+j}$, the principal axes of the positions of the levels of factor A are obtained by the following diagonalization process:

$$\mathbf{Y}_{\mathrm{A}}^{t}\mathbf{W}_{\mathrm{A}}\mathbf{Y}_{\mathrm{A}}=\mathbf{V\Phi V}^{t}$$

V, the eigenvector matrix, contains the coordinates of the principal axes of the positions of the levels of factor A on the axes of the space of DPCoA. The coordinates of the levels of factor A on these new axes are $\mathbf{Y}_{\mathrm{A}}\mathbf{V}$, the coordinates of the species $\mathbf{XV}$ and the re-centred positions of the communities are $\mathbf{P}_{\mathbf{U}_{\mathrm{B}}}^{\perp}\mathbf{Y}_{\mathrm{C}}\mathbf{V}$.

Crossed-DPCoA version 2

The projection in the orthogonal complement of the space generated by the levels of factor B is $\mathbf{\Pi}_{\mathrm{B}^{\perp}}=\mathbf{I}_{v}-\mathbf{Y}_{\mathrm{B}}^{t}\left(\mathbf{Y}_{\mathrm{B}}\mathbf{Y}_{\mathrm{B}}^{t}\right)^{-1}\mathbf{Y}_{\mathrm{B}}$ (Text S1 Proof 2), where $\mathbf{I}_{v}$ is the $v\times v$ identity matrix.

In this orthogonal complement, the coordinates of the species, the levels of factor A and the communities are in the rows of the matrices $\mathbf{X\Pi}_{\mathrm{B}^{\perp}}$, $\mathbf{Y}_{\mathrm{A}}\mathbf{\Pi}_{\mathrm{B}^{\perp}}$, $\mathbf{Y}_{\mathrm{C}}\mathbf{\Pi}_{\mathrm{B}^{\perp}}$, respectively. The principal axes of the positions of the levels of factor A are given by the general singular value decomposition of $\left(\mathbf{Y}_{\mathrm{A}}\mathbf{\Pi}_{\mathrm{B}^{\perp}},\mathbf{I}_{v},\mathbf{W}_{\mathrm{A}}\right)$: i.e. $\mathbf{\Pi}_{\mathrm{B}^{\perp}}^{t}\mathbf{Y}_{\mathrm{A}}^{t}\mathbf{W}_{\mathrm{A}}\mathbf{Y}_{\mathrm{A}}\mathbf{\Pi}_{\mathrm{B}^{\perp}}=\mathbf{U}_{\mathrm{A}}\mathbf{\Psi}\mathbf{U}_{\mathrm{A}}^{t}$, where $\mathbf{W}_{\mathrm{A}}$ is $diag\left(\mathbf{w}_{\mathrm{A}}\right)$, the diagonal matrix with the weights associated with the levels of factor A, $\mathbf{U}_{\mathrm{A}}$ are eigenvectors, and $\mathbf{\Psi}$ is the diagonal matrix of eigenvalues. $\mathbf{U}_{\mathrm{A}}$ contains the coordinates of the principal axes of the positions of the levels of factor A in the orthogonal complement of the space generated by the levels of factor B.

Accordingly, the final coordinates of the species, the levels of factor A and the communities are given in $\mathbf{X}_{/\mathrm{B}}=\mathbf{X\Pi}_{\mathrm{B}^{\perp}}\mathbf{U}_{\mathrm{A}}$, $\mathbf{Y}_{\mathrm{A/B}}=\mathbf{Y}_{\mathrm{A}}\mathbf{\Pi}_{\mathrm{B}^{\perp}}\mathbf{U}_{\mathrm{A}}$, $\mathbf{Y}_{\mathrm{C/B}}=\mathbf{Y}_{\mathrm{C}}\mathbf{\Pi}_{\mathrm{B}^{\perp}}\mathbf{U}_{\mathrm{A}}$. All these coordinates are centred according to their associated weights (Text S1 Proofs 1 and 3).

Now we will provide simple examples of the projection on the orthogonal complement of the space generated by the levels of factor B. To obtain examples that can be visualised, we defined them in 2 dimensions. Two levels of B only will be considered so that only a 1-dimensional axis is generated by factor B. Let G_X be the space generated by the species' points, G_B the space generated by the points associated with the levels of factor B, and $G_{\mathrm{B}}^{\perp}$ the

orthogonal complement to G_B, then $G_X = G_B \oplus G_B^{\perp}$. We consider that G_X has 2 dimensions, G_B 1 dimension and $G_B^{\perp}$ 1 dimension.

In these simple examples, we will only provide the positions of the communities (A1B1, A1B2, A2B1, A2B2, A3B1, A3B2), levels of factor A (A1, A2, A3) and levels of factor B (B1, B2). The position of A1 is at the centroid of A1B1, A1B2, and so on for A2, and A3. Similarly, the position of B1 is at the centroid of A1B1, A2B1, A3B1, and the position of B2 at the centroid of A1B2, A2B2, A3B2.

In each figure below, the panel on the left is the theoretical space of DPCoA, with the triangle indicating the centre of the space. The axes of this space have not been indicated because we are more interested in the principal axis of the levels of factor B: blue axis (which generates space G_B). The red axis is orthogonal to the blue one and generates $G_B^{\perp}$. All points are projected on this red axis in the right panel of each figure.

Even if in reality, more dimensions will be necessary to define G_X, G_B, and $G_B^{\perp}$, these simple examples show how any diversity patterns due to factor A only or to the interaction A×B that are in the same direction as that of the diversity pattern generated by factor B is eliminated.

Example 1: The line generated by A1 and A2 is parallel to that generated by B1 and B2, so that A1 and A2 have the same position in $G_B^{\perp}$.

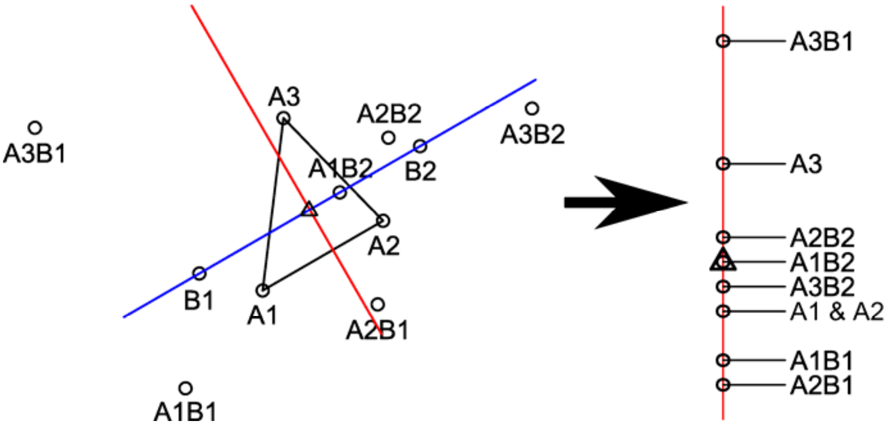

Example 2: Most of the inertia associated with the levels of factor A in G_X is conserved in $G_B^{\perp}$

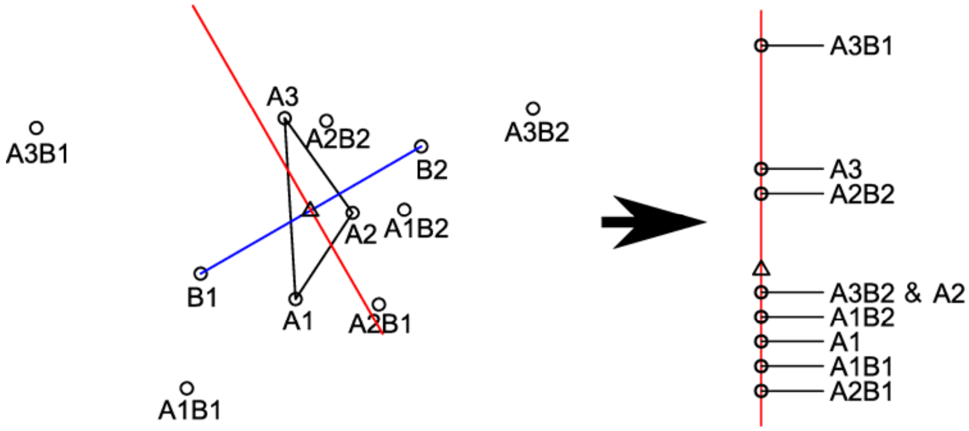

Example 3: None of the inertia associated with the levels of factor A in G_X is conserved in $G_B^\perp$. The inertia of communities' points in $G_B^\perp$ is *SS*(A,B), the interaction component.

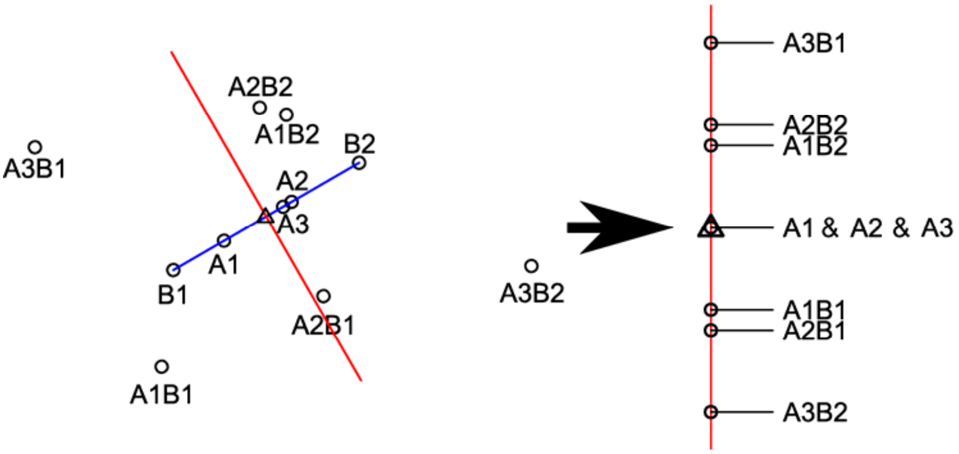

Example 4: All of the inertia associated with the levels of factor A in G_X is conserved in $G_B^\perp$.

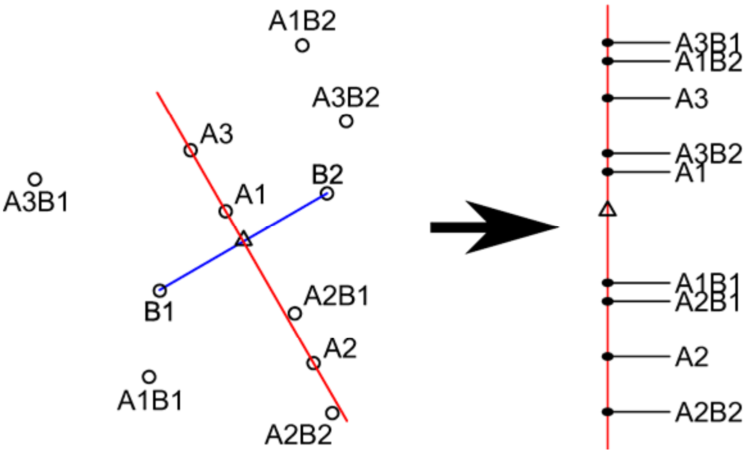

Situations where $w_{ij} \neq w_{i+}w_{+j}$

The algorithms used for the rapid analysis of the main effect of B and for crossed DPCoA version 2 are unchanged.

Regarding crossed-DPCoA version 1, if the importance values given to the levels of factor A and B are not independent so that $w_{ij} \neq w_{i+}w_{+j}$, then the position of the levels of factor A are also modified by the centring process. The modified positions are

$$\mathbf{p}_{i+}^t\mathbf{X} - \sum_{j=1}^{m} \frac{w_{ij}}{w_{i+}} \mathbf{p}_{+j}^t\mathbf{X} = \mathbf{p}_{i+}^t\mathbf{X} - \sum_{i=1}^{r}\sum_{j=1}^{m} \frac{w_{ij}}{w_{i+}} \frac{w_{ij}}{w_{+j}} \mathbf{p}_{ij}^t\mathbf{X}$$

Nevertheless their centroid is still the centre of the space:

$$\begin{aligned}\sum_{i=1}^{r} w_{i+}\left[\mathbf{p}_{i+}^t\mathbf{X} - \sum_{j=1}^{m}\frac{w_{ij}}{w_{i+}}\mathbf{p}_{+j}^t\mathbf{X}\right] &= \sum_{i=1}^{r} w_{i+}\mathbf{p}_{i+}^t\mathbf{X} - \sum_{i=1}^{r}\sum_{j=1}^{m} w_{ij}\mathbf{p}_{+j}^t\mathbf{X} \\ &= \sum_{i=1}^{r} w_{i+}\mathbf{p}_{i+}^t\mathbf{X} - \sum_{j=1}^{m} w_{+j}\mathbf{p}_{+j}^t\mathbf{X} \\ &= \mathbf{0}\end{aligned}$$

Analysing the principal axes of the new coordinates of the levels of factor A would not be optimal. A better solution was introduced in the context of conditional redundancy analysis [1], conditional canonical correspondence analysis [2].

Let $\mathbf{U}_A$ be the matrix with communities as rows and levels of factor A as columns. The entry at the ith row and j^{th} column contains 1 if the community i is associated with the jth level of factor A and 0 otherwise. This matrix is the disjunctive matrix associated with factor A.
Let $\mathbf{U}_B$ be the matrix with communities as rows and levels of factor B as columns. The entry at the ith row and j^{th} column contains 1 if the community i is associated with the jth level of factor B and 0 otherwise. This matrix is the disjunctive matrix associated with factor B.
As shown above, the projector on the orthogonal complement of the subspace generated by $\mathbf{U}_B$ is

$$\mathbf{P}_{\mathbf{U}_B}^{\perp} = \mathbf{I}_{rm} - \mathbf{U}_B\left(\mathbf{U}_B^t\mathbf{W}_C\mathbf{U}_B\right)^{-1}\mathbf{U}_B^t\mathbf{W}_C$$

the projector on the subspace generated by $\mathbf{U}_A$ is

$$\mathbf{P}_{\mathbf{U}_A} = \mathbf{U}_A\left(\mathbf{U}_A^t\mathbf{W}_C\mathbf{U}_A\right)^{-1}\mathbf{U}_A^t\mathbf{W}_C$$

Let (A) be the space generated by $\mathbf{U}_A$ and $(A)^{\perp}$ its complements. Similarly (B) is the space generated by $\mathbf{U}_B$ and $(B)^{\perp}$ its complement. Let (A+B) be the space generated by $\mathbf{U}_{A+B}$, where $\mathbf{U}_{A+B}$ is the matrix with communities as rows obtained as follows: the first column contains only ones, the next columns are all but one column of $\mathbf{U}_A$ and the last columns contain all but one column of $\mathbf{U}_B$. In the case of orthogonal factors, $(A+B) = (A) \oplus (B)$ so that the orthogonal complement of (B) in (A+B) is (A). When all combinations of the factors are orthogonal and when $w_{ij}=w_{i+}w_{+j}$, then the two factors are orthogonal.

However, when the two factors are not orthogonal then $(A+B) = (A/B) \oplus (B)$. The subspace of (A+B) named (A/B) is generated by the matrix $\mathbf{P}_{\mathbf{U}_B}^{\perp}\mathbf{U}_A$. The projector on this subspace is

$$\mathbf{P}_{(A/B)} = \mathbf{P}_{\mathbf{U}_B}^{\perp}\mathbf{U}_A\left(\mathbf{U}_A^t\mathbf{W}_C\mathbf{P}_{\mathbf{U}_B}^{\perp}\mathbf{U}_A\right)^{-1}\mathbf{U}_A^t\mathbf{W}_C\mathbf{P}_{\mathbf{U}_B}^{\perp}$$

Starting with the space of the DPCoA (see Text S1), all communities' points can be projected on (A/B) leading to the matrix $\mathbf{P}_{(A/B)}\mathbf{Y}_C$ that contains the new coordinates of the communities on the axes of this new space.

Then the principal component analysis of $\mathbf{P}_{(A/B)}\mathbf{Y}_C$ weighted by $\mathbf{W}_C$ provides a space where the partial main effect of A can be analysed. The sum of all eigenvalues of this principal component analysis will be lower or equal to $SS(A)$. It would be equal to $SS(A)$ if $\mathbf{P}_{(A/B)}=\mathbf{P}_{(A)}$ so if A and B are orthogonal factors.

Dealing with repetitions

When several plots have been sampled within each community, each plot is associated with a level of factor A and a level of factor B in accordance with the community to which it belongs.

Let p_{ijkl} the proportions of species l in the plot k of community ij. Let w_{ijk} be an importance value given to the plot k of community ij so that $w_{ij} = \sum_{k=1}^{n_{ij}} w_{ijk}$, where n_{ij} is the number of plots analysed within community ij. The proportion of species l in community ij is

$$p_{ij+l} = \sum_{k=1}^{n_{ij}} \frac{w_{ijk}}{w_{ij}} p_{ijkl}$$

The previous analyses can thus be performed by replacing p_{ijk} with these p_{ij+l} values.
In that case the decomposition of the quadratic entropy is still

$$SST=SSW+\text{SS(C)}$$

with SS(C)=SS(A)+SS(B)+SS(A,B).
But SSW can be divided into $SSWP$+SS(P), where $SSWP$ is the diversity within-plots and SS(P) is the diversity among points but within communities. Repetitions thus permit a further decomposition of the component SSW and they might provide a better estimation of the proportions of species within communities depending on the sampling scheme and the structure of the communities analysed.

Points for plots can be added to the space of DPCoA. For instance, with **X** the matrix of species coordinates and $\mathbf{p}_{ijk} = \left(p_{ijk1},...,p_{ijkl},...,p_{ijkS}\right)$, the coordinates of the position of plot k from community ij are in the vector

$$\mathbf{p}_{ijk}^t\mathbf{X}$$

They are centred for the weights w_{ijk}:

$$\sum_{i=1}^{r}\sum_{j=1}^{m}\sum_{k=1}^{n_{ij}} w_{ijk}\mathbf{p}_{ijk}^t\mathbf{X} = \sum_{i=1}^{r}\sum_{j=1}^{m} w_{ij}\sum_{k=1}^{n_{ij}}\frac{w_{ijk}}{w_{ij}}\mathbf{p}_{ijk}^t\mathbf{X}$$

$$= \sum_{i=1}^{r}\sum_{j=1}^{m} w_{ij}\mathbf{p}_{ij}^t\mathbf{X} = \mathbf{0}_v^t$$

These points can be projected on all subspaces of the space of DPCoA defined in the paragraphs above.

Unbalanced schemes: when some combinations of the interacting factors are missing

Independent weights for factor A and B are not possible if some of the combinations between the two factors are missing because if $w_{ij}=w_{i+}w_{+j}$ and one combination ij is missing then $\sum_{ij \text{ available}} w_{ij} \neq 1$.

The w_{ij} must thus be first defined so that $\sum_{ij \text{ available}} w_{ij} = 1$. Then,

$w_{i+} = \sum_{j \text{ available}} w_{ij}$, is the weight attributed to level i of factor A.

$w_{+j} = \sum_{i \text{ available}} w_{ij}$ is the weight attributed to level j of factor B.

$p_{++k} = \sum_{ij \text{ available}} w_{ij}p_{ijk}$ is the weight attributed to species k, where p_{ijk} is the proportion of species k in the community ij.

$\mathbf{p}_{i+} = \sum_{j \text{ available}} w_{ij}\mathbf{p}_{ij} / w_{i+}$ is the vector of species proportions associated with level i of factor A (A definition of p_{ij} is given at the beginning of this document).

$\mathbf{p}_{+j} = \sum_{i \text{ available}} w_{ij}\mathbf{p}_{ij} / w_{+j}$ is the vector of species proportions associated with level j of factor B.

$\mathbf{p}_{++} = \sum_{ij \text{ available}} w_{ij}\mathbf{p}_{ij}$ is the vector of species proportions over the whole data set.

The remaining notations are unchanged.

In that case even the main effects of factor A and B are clearly not independent. If an analysis of the data is still required then it should follow the situation where $w_{ij} \neq w_{i+}w_{+j}$ (see above).

Other propositions and discussions

The situation were $w_{ij}=w_{i+}w_{+j}$ (the situation chosen in the main text) offers more possibilities than when $w_{ij} \neq w_{i+}w_{+j}$.

In crossed-DPCoA version 1 and 2, when $w_{ij}=w_{i+}w_{+j}$ the crossed-DPCoA versions are both structured in two successive steps. At the first step, all points are projected on a space orthogonal to the main effect of B. In version 1, the main effect is described by the positions of the levels of factor B in the extended space of DPCoA. In version 2, it is described by the subspace generated by these positions in the extended space of the DPCoA. Version 2 thus considers more information about the main effect of B than version 1.

When the projection is done, the new space contains species' points, communities' points and points for the levels of factor A. The points for the levels of factor B, all now located at the centre of the new space, are discarded. We have chosen to analyse the principal axes of factor A and to project all points on these principal axes. These principal axes best distinguish the positions of the level of factor A (the main effect of A) but the communities' point on these axes inform about how the interaction between A and B add to the main effect to reveal the total effect of factor A (main effect + interaction with B).

Another solution would be to analyse the principal axes of the communities' points (which represent both the main effect of A and the interaction with B). In our case study the two solutions provided similar results.

1. Sabatier R, Lebreton JD, Chessel D (1989) Principal component analysis with instrumental variables as a tool for modelling composition data. In: Coppi R, Bolasco S, editors. Multiway data analysis: Elsevier Science Publishers B.V., North-Holland. pp. 341-352.
2. Ter Braak CJF (1987) Unimodal models to relate species to environment. Agricultural Mathematics Groups. Wageningen, the Netherlands: Box, Box 100, NL-6700 AC. pp. 83-89.
