# Supplementary material for: A New Technique for Analysing Interacting Factors Affecting Biodiversity Patterns: Crossed-DPCoA
Source: PLoS One. 2013 Jan 24;8(1):e54530. doi: 10.1371/journal.pone.0054530 (PMC3554745; doi:10.1371/journal.pone.0054530)
Supplement: Text S4 — Manual for R scripts. This appendix uses data available in Dataset S1 and R scripts available in Text S3. (PDF) [file pone.0054530.s005.pdf]

Text S4. Manual for R scripts

R script, see http://cran.r-project.org/.

This program is free software: you can redistribute it and/or modify it under the terms of the GNU General Public License http://www.gnu.org/licenses/.

The program was checked with R version 2.5.1. In the future, the first author may be contacted for updated version of the scripts.

While due care has been taken and it is believed accurate, its use is solely the responsibilities of the user.

Data and scripts will be available in the future in the package ade4 to assure changes required by the continuous improvements of R environment.

The package ade4 must be loaded before using the functions. Write in your R console:

```
library(ade4)
```

Three functions have been written: `crossdpcoa_maineffect`, `crossdpcoa_version1`, `crossdpcoa_version2`

The function `crossdpcoa_maineffect` obtains the space of DPCoA and the principal axes of the positions of the levels of one of the factors in this space and project species' points on these principal axes. It has the following usage:

```
crossdpcoa_maineffect(df, facA, facB, dis = NULL, scannf = TRUE, nf = 2,
      w = "classic", tol = 1e-07)
```

The main effect of the factor `facA` is analysed by this process.

The function `crossdpcoa_version1` performs version 1 of the crossed DPCoA. It has the following usage:

```
crossdpcoa_version1(df, facA, facB, dis = NULL, scannf = TRUE, nf = 2,
      w = "classic", tol = 1e-07)
```

The effect of factor `facA` given factor `facB` is analysed by this process.

The function `crossdpcoa_version2` performs version 2 of the crossed DPCoA. It has the following usage:

```
crossdpcoa_version2(df, facA, facB, dis = NULL, scannf = TRUE, nf = 2,
      w = "classic", tol = 1e-07)
```

The effect of factor `facA` given factor `facB` is analysed by this process.

The parameters in all these functions are defined as follows:

Parameter	Explanation
`df`	a data frame of 0/1 or nonnegative values (e.g. in our case study, a species × communities data frame)

`facA`	a factor with the same length as the number of columns in df (e.g. in our case study, the five locations Burgundy, Poland, Provence, Corsica and Algeria)
`facB`	a factor with the same length as the number of columns in df (e.g. in our case study, the six seral stages)
`dis`	an object of class '`dist`' that contains the distances among species (in our case studies these are phylogenetic distances). If `NULL` equidistances are used among species.
`scannf`	a logical value indicating whether the screeplot should be displayed.
`nf`	if `scannf` FALSE, an integer indicating the number of kept axes
`w`	either a character or a numeric vector of positive values that indicates how the columns of df (e.g. in our case study the communities) should be weighted. If `w="classic"`, the weights are defined from the sum of the values in each column (e.g. sum of all species abundances within a community). If `w="independence"`, then the weight attributed to a column is the product of the weight attributed to a level of factor A with the weight attributed to a level of factor B. See details below.
`tol`	a tolerance threshold: a value less than tol times is considered as null.

The functions `crossdpcoa_maineffect, crossdpcoa_version1 and crossdpcoa_version2` return a list containing the used information for computing the crossed-DPCoA:

**Parameter**	**Explanation**
`l1`	coordinates of the rows of df (in our case study, the species)
`l2`	coordinates of the levels of factor A (in our case study, the locations)
`l3`	(for functions crossdpcoa_version1 and crossdpcoa_version2 only) coordinates of the columns of df (in our case study, the communities)
`eig`	the eigenvalues
`lwX`	the weights attributed to the species
`lwA`	the weights attributed to the levels of factor A
`lwB`	the weights attributed to the levels of factor B
`lwC`	the weights attributed to the communities
`div`	a numeric vector with the decomposition of Rao's quadratic diversity.
`call`	the call function

####################

Details for argument w:

If `w="classic"`, the following weights are used:
Denote a_{ijk} the estimated abundance might be based on density, percentage cover (for plants), biomass or number of individuals, $a_{ij+} = \sum_{k=1}^{S} a_{ijk}$ and $a_{+++} = \sum_{i=1}^{r}\sum_{j=1}^{m}\sum_{k=1}^{S} a_{ijk}$, and consider that the proportion of species *k* in community *ij* is defined as $p_{ijk} = a_{ijk} / a_{ij+}$. Then, the weight of community *ij* is defined as $w_{ij} = a_{ij+} / a_{+++}$. The weights of the factor levels are defined as follows: $w_{i+} = \sum_{j=1}^{m} w_{ij}$ is the weight attributed to the level *i* of factor A and $w_{+j} = \sum_{i=1}^{r} w_{ij}$ is the weight attributed to the level *j* of factor B.

If `w="independence"`, the weights of the levels of factor A and those of the levels of factor B are defined as with `w="classic"`. However the weights of the communities are then defined as $w_{ij} = w_{i+}w_{+j}$.

If `w` is a numeric vector of nonnegative values, then the vector is standardized as w/sum(w) to obtain the weights of the communities. These values should be in the same orders as the columns in df.
#####################

Application:

First we must load the functions and the data available in Text S3 and Dataset S1. Use the function '`source`' to load the functions and the function '`load`' to load the data.

The data set consists in a list of four objects. The name of the list is BirdData. The names of the four objects are:

```
names(BirdData)
[1] "fau"  "tre"  "facA" "facB"
```

`fau` is a data frame with species as rows, communities as columns and densities as entries.

The names of the communities start with the first three letters of the location and ends with the seral stage number:

```
names(BirdData$fau)
[1] "Alg1" "Alg2" "Alg3" "Alg4" "Alg5" "Alg6" "Bur1"
 [8] "Bur2" "Bur3" "Bur4" "Bur5" "Bur6" "Cor1" "Cor2"
[15] "Cor3" "Cor4" "Cor5" "Cor6" "Pol1" "Pol2" "Pol3"
[22] "Pol4" "Pol5" "Pol6" "Pro1" "Pro2" "Pro3" "Pro4"
[29] "Pro5" "Pro6"
```

`tre` is a character. It contains the phylogeny with a newick format.

`facA` and `facB` are factors:

```
BirdData$facA
[1]  Alg Alg Alg Alg Alg Alg Bur Bur Bur Bur Bur
[12] Bur Cor Cor Cor Cor Cor Cor Pol Pol Pol Pol
[23] Pol Pol Pro Pro Pro Pro Pro Pro
Levels: Alg Bur Cor Pol Pro
```

The locations are Alg = Algeria, Bur = Burgundy, Cor = Corsica Island, Pol = Poland, Pro = Provence.

```
BirdData$facB
 [1] S1 S2 S3 S4 S5 S6 S1 S2 S3 S4 S5 S6 S1 S2 S3
[16] S4 S5 S6 S1 S2 S3 S4 S5 S6 S1 S2 S3 S4 S5 S6
Levels: S1 S2 S3 S4 S5 S6
```

The seral stages are numbered from S1 to S6.

The phylogenetic tree is obtained with

```
phy <- newick2phylog(BirdData$tre)
```

The phylogenetic distances among species are obtained with

```
phydis <- phy$Wdist
```

We take care that species in the data frame are in the same order as species in the tips of the phylogeny:

```
attach(BirdData)
fau <- fau[names(phy$leaves), ]
```

Analysis of the main effect of B:

The main effect of B was analysed as follows:

```
cd_mainB <- crossdpcoa_maineffect(fau, facB, facA, phydis, w=rep(1/30, 30),
scannf = FALSE)
```

eigenvalue bar plot:

```
barplot(cd_mainB$eig)
```

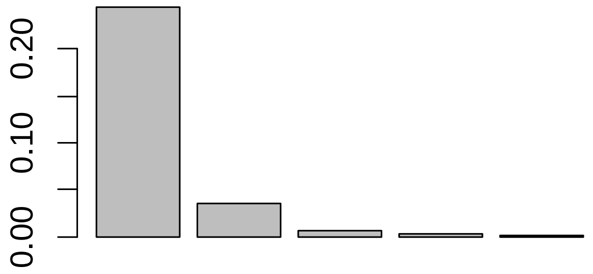

Proportion of *SS*(B) expressed by the two first axes:

```
cd_mainB$eig[1:2]/sum(cd_mainB$eig)
[1] 0.8405189 0.1232833
```

The main effect is mostly reflected by the first axis.

Positions of the levels of factor B on its principal axes:

```
s.label(cd_mainB$l2)
```

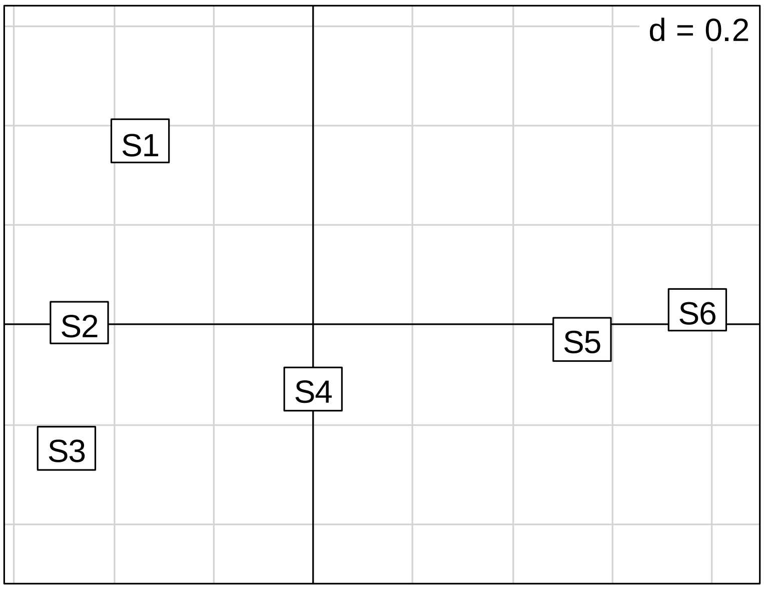

The value of ‘d’ provides the scale

The coordinates of the species on the same axes can be displayed in front of the phylogeny:

```
dotchart.phylog(phy, cd_mainB$l1, scaling=FALSE, yjoining=0)
```

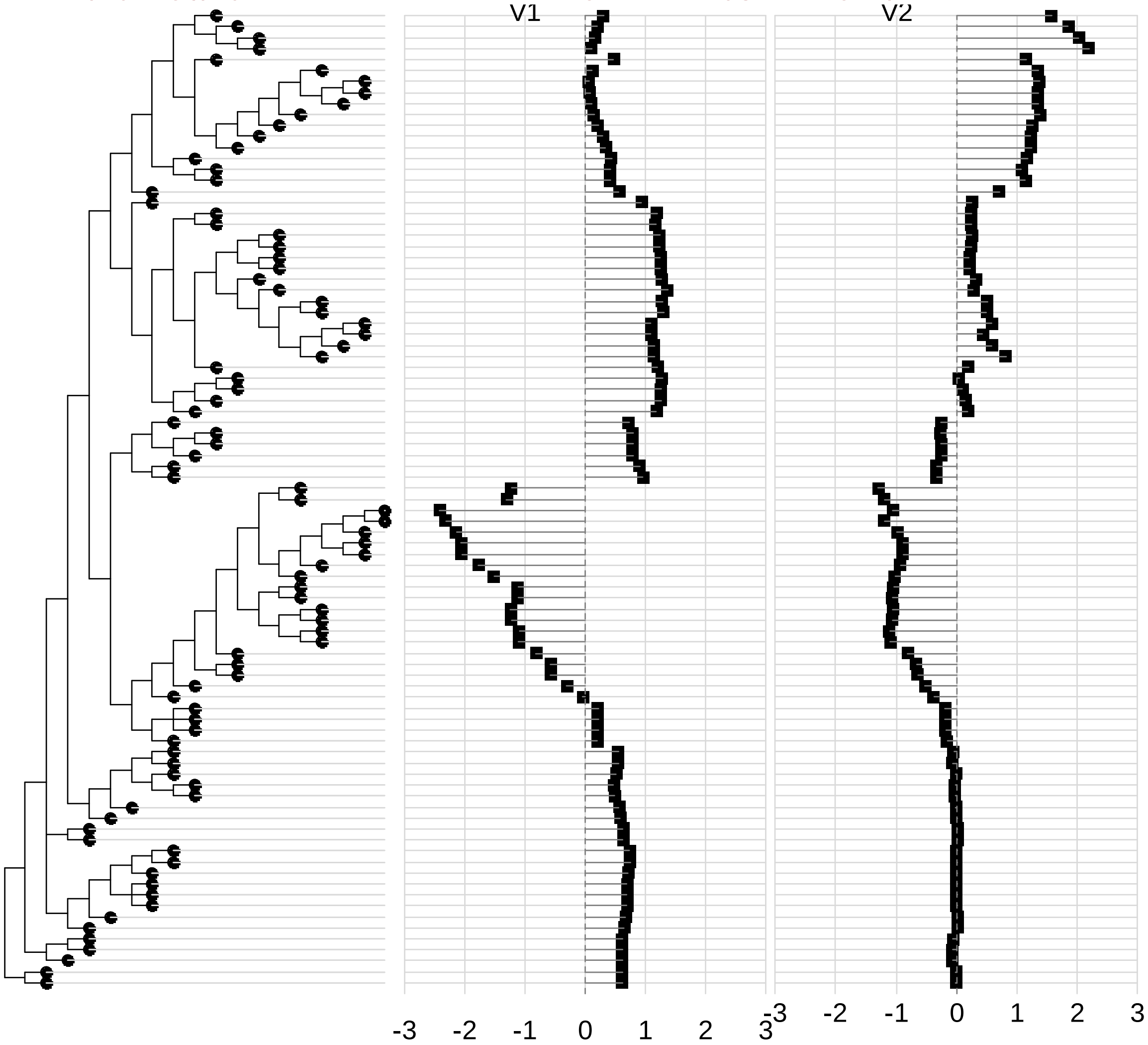

The two next analyses explore of the effects of the location on the phylogenetic composition of the communities conditional to the seral stages.

Crossed DPCoA Version 1

Crossed DPCoA version 1 can now be performed as follows:

```
cd_v1 <- crossdpcoa_version1(fau, facA, facB, phydis, w=rep(1/30, 30),
scannf = FALSE)
```

Proportion of *SS*(A) expressed by the two first axes:

```
cd_v1$eig[1:2]/sum(cd_v1$eig)
[1] 0.6338212 0.1654986
```

To view the positions of the locations on the first two axes, write:

```
s.label(cd_v1$l2)
```

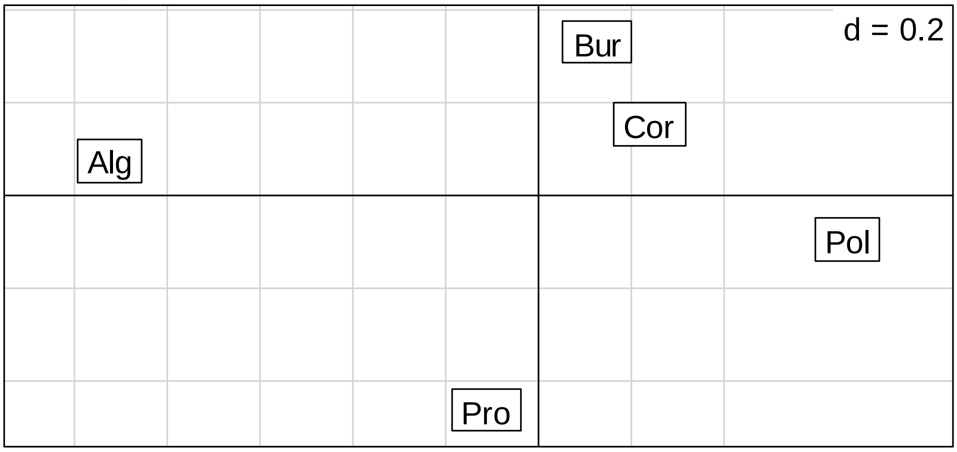

To view the positions of all communities on the first two axes, write:

```
s.label(cd_v1$l3)
```

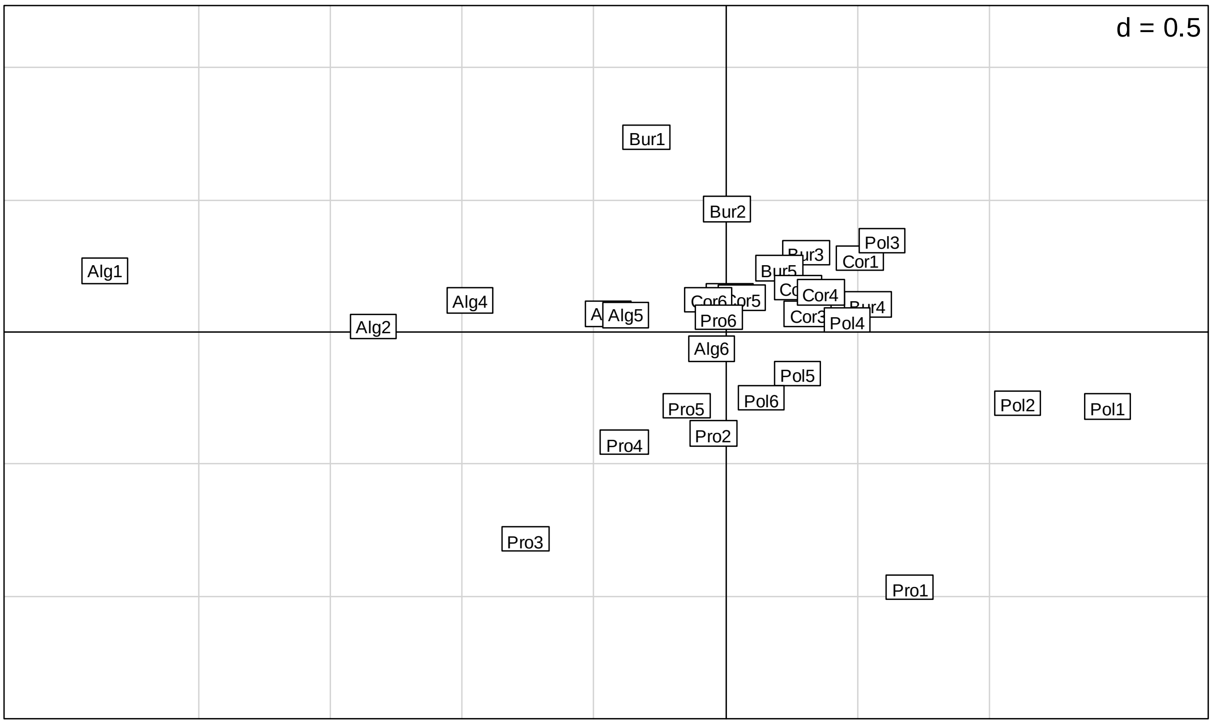

To view the positions of the species on the first two axes in front of the phylogeny, write:

```
dotchart.phylog(phy, cd_v1$l1, scaling=FALSE, yjoining=0)
```

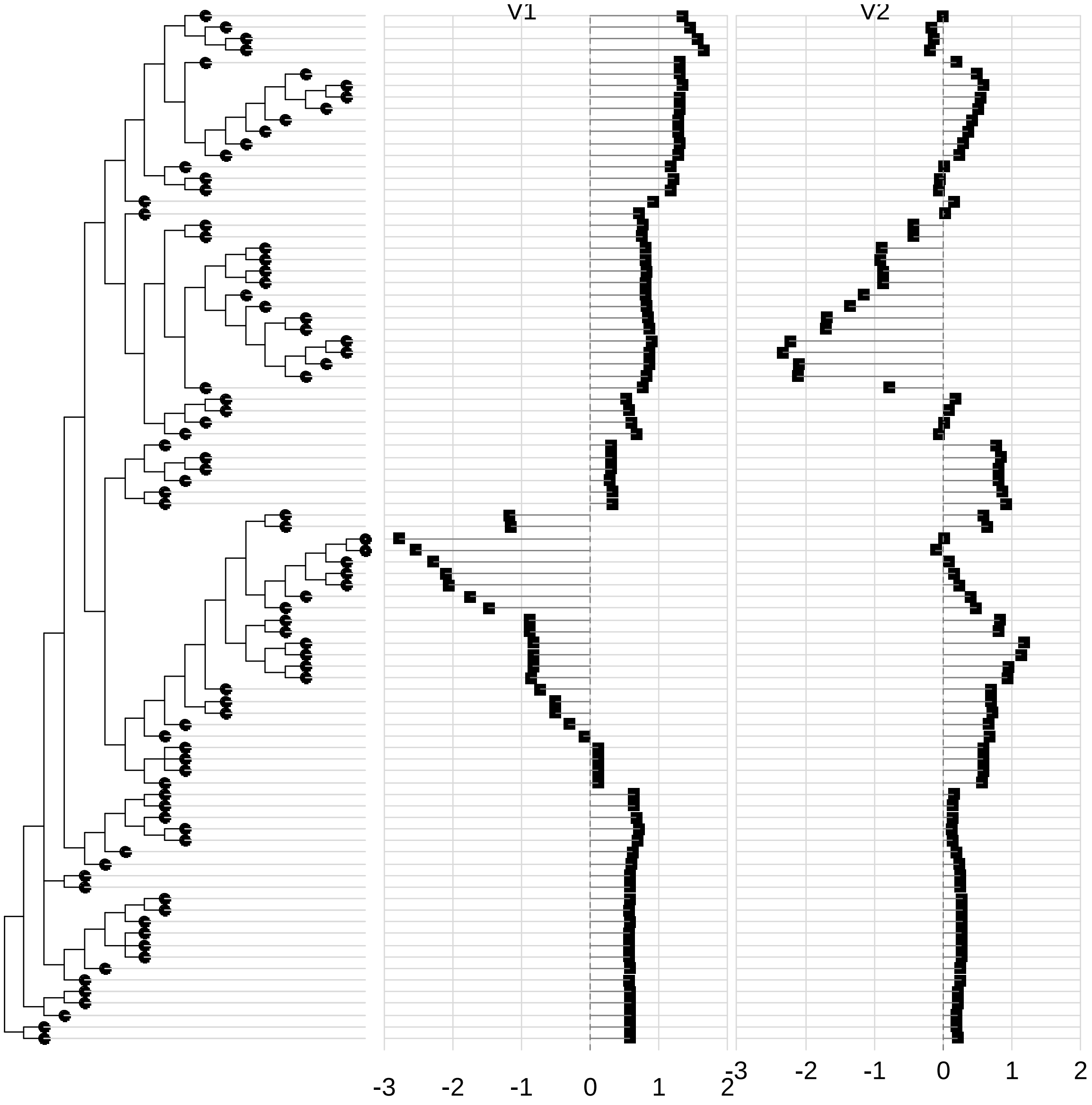

Crossed DPCoA Version 2

Crossed DPCoA version 2 can now be performed as follows:

```
cd_v2 <- crossdpcoa_version2(fau, facA, facB, phydis, w=rep(1/30, 30),
scannf = FALSE)
```

Proportion of variation among levels of factor A in the subspace orthogonal to the principal axes of B expressed by the two first axes:

```
cd_v2$eig[1:2]/sum(cd_v2$eig)
[1] 0.5734640 0.1966385
```

To view the positions of the locations on the first two axes, write:

```
s.label(cd_v2$l2)
```

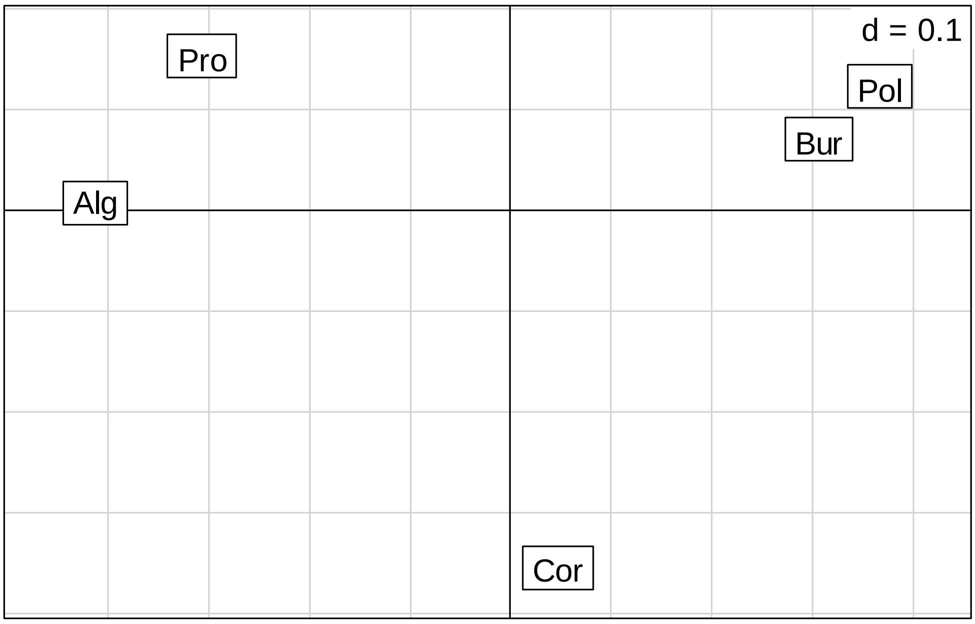

To view the positions of all communities on the first two axes, write:

```
s.label(cd_v2$l3)
```

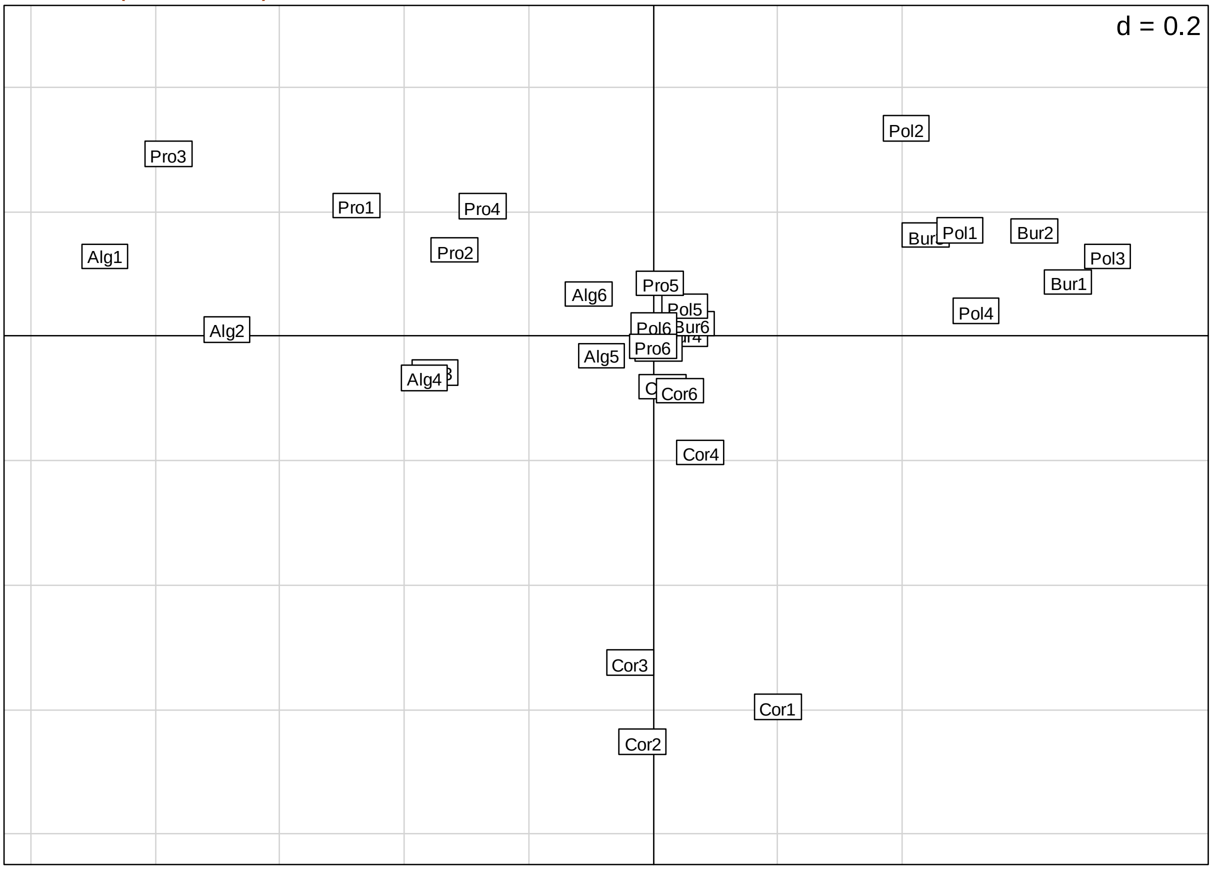

To view the positions of the species on the first two axes in front of the phylogeny, write:

```
dotchart.phylog(phy, cd_v2$l1, scaling=FALSE, yjoining=0)
```

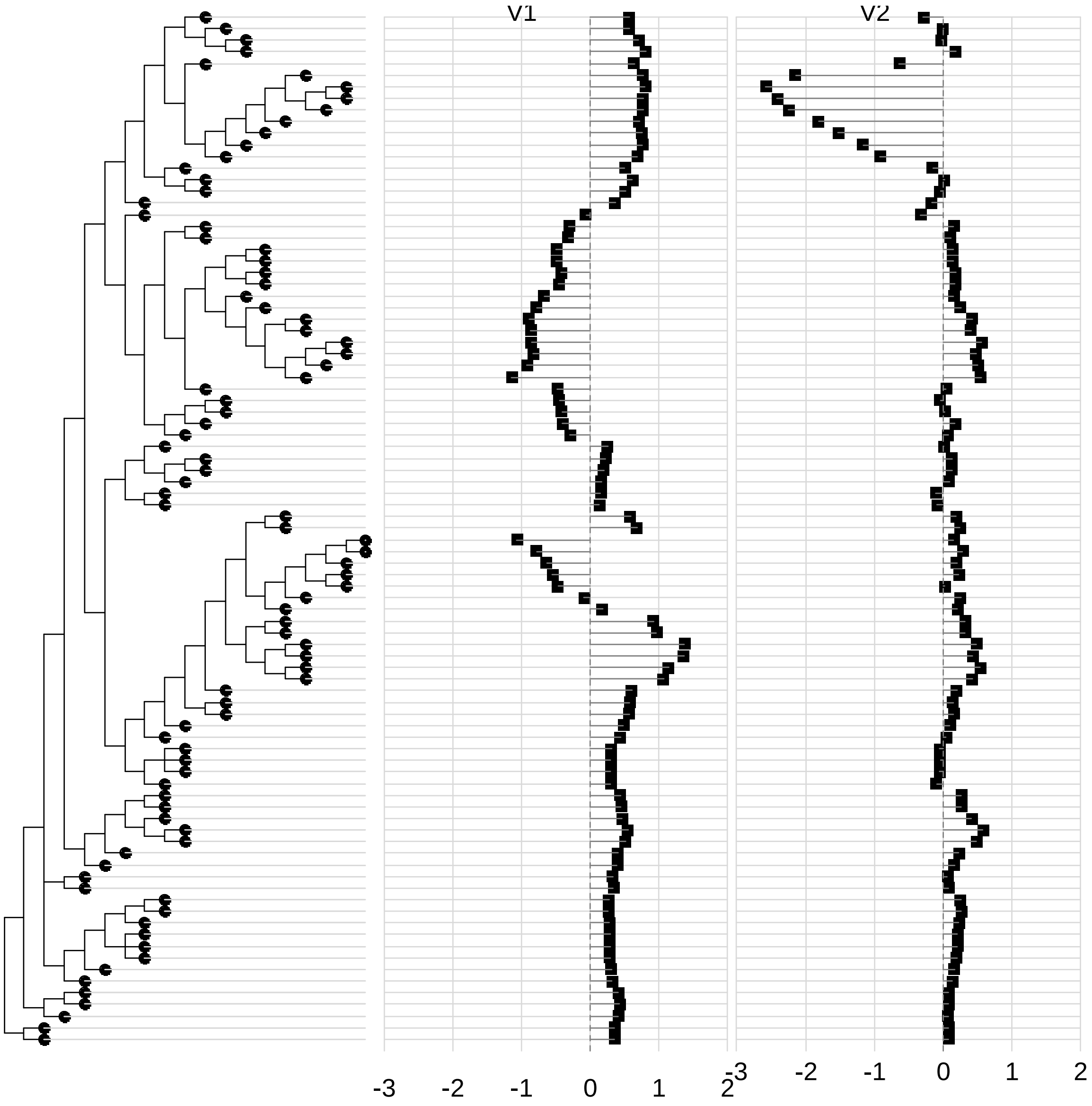
V1
V2
-3
-2
-1
0
1
2
-3
-2
-1
0
1
2
