# Supplementary material for: A New Technique for Analysing Interacting Factors Affecting Biodiversity Patterns: Crossed-DPCoA
Source: PLoS One. 2013 Jan 24;8(1):e54530. doi: 10.1371/journal.pone.0054530 (PMC3554745; doi:10.1371/journal.pone.0054530)
Supplement: Text S5 — Species whose positions in the phylogeny were not defined by Davis. This appendix contains details on the establishment of the phylogeny. (PDF) [file pone.0054530.s006.pdf]

Text S5 – Species whose positions in the phylogeny were not defined by Davis

14 species out of 89 were not positioned in [1].

The following species were placed according to [2]:
Columba oenas, Dendrocopos medius, Dryocopus martius, Galerida cristata, G. theklae, Locustella naevia, Lullula arborea, Luscinia luscinia, L. megarhynchos, Turdus viscivorus

The following species was placed according to [3]:
Emberiza hortulana

Three species have been placed as follows:

- *Corvus cornix* has been placed based on *Corvus corone* the position of which was given in [1];
- *Oenanthe hispanica* has been placed based on the position of *Oenanthe oenanthe* in [2];
- *Picus vaillantii* has been placed with *P. canus* and *P. viridis.*

References:

[1] Davis KE (2008) Reweaving the tapestry: a supertree of birds. PhD Thesis. University of Glasgow, Glasgow, U.K. http://linnaeus.zoology.gla.ac.uk/~rpage/birdsupertree/index.php

[2] Møller AP (2006) Sociality, age at first reproduction and senescence: comparative analyses of birds. J evolution biol 19: 682-689

[3] Alström P, Olsson U, Lei F, Wang H-T, Gao W, Sundberg P (2008) Phylogeny and classification of the Old World Emberizini (Aves, Passeriformes). Mol Phylogenet Evol 47: 960-973.
