# Supplementary material for: A New Technique for Analysing Interacting Factors Affecting Biodiversity Patterns: Crossed-DPCoA
Source: PLoS One. 2013 Jan 24;8(1):e54530. doi: 10.1371/journal.pone.0054530 (PMC3554745; doi:10.1371/journal.pone.0054530)
Supplement: Text S6 — Bird taxonomy. (PDF) [file pone.0054530.s007.pdf]

Text S6 – Taxonomy used for the 89 bird species.

Species are given in the same order as in Fig. 3 of the main text.

	**Family**	**Order**
Emberiza calandra	Emberizidae	Passeriformes
Emberiza hortulana	Emberizidae	Passeriformes
Emberiza cirlus	Emberizidae	Passeriformes
Emberiza citrinella	Emberizidae	Passeriformes
Fringilla coelebs	Fringillidae	Passeriformes
Carduelis carduelis	Fringillidae	Passeriformes
Carduelis citrinella	Fringillidae	Passeriformes
Serinus serinus	Fringillidae	Passeriformes
Serinus canaria	Fringillidae	Passeriformes
Carduelis cannabina	Fringillidae	Passeriformes
Carduelis chloris	Fringillidae	Passeriformes
Coccothraustes coccothraustes	Fringillidae	Passeriformes
Pyrrhula pyrrhula	Fringillidae	Passeriformes
Motacilla alba	Motacillidae	Passeriformes
Anthus trivialis	Motacillidae	Passeriformes
Anthus campestris	Motacillidae	Passeriformes
Prunella modularis	Prunellidae	Passeriformes
Regulus ignicapillus	Regulidae	Passeriformes
Sturnus vulgaris	Sturnidae	Passeriformes
Sturnus unicolor	Sturnidae	Passeriformes
Saxicola torquata	Turdidae	Passeriformes
Saxicola rubetra	Turdidae	Passeriformes
Turdus philomelos	Turdidae	Passeriformes
Turdus viscivorus	Turdidae	Passeriformes
Muscicapa striata	Muscicapidae	Passeriformes
Erithacus rubecula	Muscicapidae	Passeriformes
Ficedula hypoleuca	Muscicapidae	Passeriformes
Ficedula albicollis	Muscicapidae	Passeriformes
Luscinia luscinia	Muscicapidae	Passeriformes
Luscinia megarhynchos	Muscicapidae	Passeriformes
Phoenicurus phoenicurus	Muscicapidae	Passeriformes
Oenanthe hispanica	Muscicapidae	Passeriformes
Turdus merula	Turdidae	Passeriformes
Troglodytes troglodytes	Troglodytidae	Passeriformes
Certhia familiaris	Certihiidae	Passeriformes
Certhia brachydactyla	Certihiidae	Passeriformes
Sitta europaea	Sittidae	Passeriformes
Poecile montana	Paridae	Passeriformes
Poecile palustris	Paridae	Passeriformes
Lophophanes cristatus	Paridae	Passeriformes
Periparus ater	Paridae	Passeriformes
Parus major	Paridae	Passeriformes
Cyanistes caeruleus	Paridae	Passeriformes
Sylvia atricapilla	Sylviidae	Passeriformes

Sylvia borin	Sylviidae	Passeriformes
Sylvia melanocephala	Sylviidae	Passeriformes
Sylvia cantillans	Sylviidae	Passeriformes
Sylvia conspicillata	Sylviidae	Passeriformes
Sylvia undata	Sylviidae	Passeriformes
Sylvia sarda	Sylviidae	Passeriformes
Sylvia communis	Sylviidae	Passeriformes
Sylvia hortensis	Sylviidae	Passeriformes
Hippolais polyglotta	Acrocephalidae	Passeriformes
Hippolais icterina	Acrocephalidae	Passeriformes
Phylloscopus collybita	Phylloscopidae	Passeriformes
Phylloscopus trochilus	Phylloscopidae	Passeriformes
Phylloscopus sibilatrix	Phylloscopidae	Passeriformes
Phylloscopus bonelli	Phylloscopidae	Passeriformes
Cisticola juncidis	Cisticolidae	Passeriformes
Locustella fluviatilis	Megaluridae	Passeriformes
Locustella naevia	Megaluridae	Passeriformes
Pycnonotus barbatus	Pycnonotidae	Passeriformes
Aegithalos caudatus	Aegithalidae	Passeriformes
Alauda arvensis	Alaudidae	Passeriformes
Galerida cristata	Alaudidae	Passeriformes
Galerida theklae	Alaudidae	Passeriformes
Lullula arborea	Alaudidae	Passeriformes
Corvus cornix	Corvidae	Passeriformes
Garrulus glandarius	Corvidae	Passeriformes
Lanius excubitor	Laniidae	Passeriformes
Lanius collurio	Laniidae	Passeriformes
Lanius senator	Laniidae	Passeriformes
Tchagra senegala	Malaconotidae	Passeriformes
Oriolus oriolus	Oriolidae	Passeriformes
Cuculus canorus	Cuculidae	Cuculiformes
Clamator glandarius	Cuculidae	Cuculiformes
Dendrocopos medius	Picidae	Piciformes
Dendrocopos major	Picidae	Piciformes
Dendrocopos minor	Picidae	Piciformes
Picus canus	Picidae	Piciformes
Picus viridis	Picidae	Piciformes
Picus vaillantii	Picidae	Piciformes
Dryocopus martius	Picidae	Piciformes
Jynx torquilla	Picidae	Piciformes
Columba oenas	Columbidae	Columbiformes
Columba palumbus	Columbidae	Columbiformes
Streptopelia turtur	Columbidae	Columbiformes
Alectoris rufa	Phasianidae	Galliformes
Coturnix coturnix	Phasianidae	Galliformes
