# Supplementary material for: A New Technique for Analysing Interacting Factors Affecting Biodiversity Patterns: Crossed-DPCoA
Source: PLoS One. 2013 Jan 24;8(1):e54530. doi: 10.1371/journal.pone.0054530 (PMC3554745; doi:10.1371/journal.pone.0054530)
Supplement: Text S7 — Connections between crossed-DPCoA and other ordination approaches. Previously developed crossed analyses that treat species as equidistant, as with classical diversity indices are compared with crossed-DPCoA. (PDF) [file pone.0054530.s008.pdf]

Text S7. Connections between DPCoA and other ordination approaches

The connections below were established by [1] for a special choice of communities' weights. We demonstrate the connections here for any choice of communities' weights w_{ij}.

PROOF: CONNECTION BETWEEN DPCOA AND CANONICAL CORRESPONDENCE ANALYSIS

We apply here CCA to the $S \times rm$ matrix $\mathbf{P} = \left(p_{ijk} w_{ij}\right)$ with species as rows and communities as columns and to a $S \times n$ matrix $\mathbf{Z}$ with species as rows and n quantitative variables as columns. A possible algorithm for CCA of $\mathbf{P}$ and $\mathbf{Z}$ is as follows.
Let $\mathbf{W}_S$ be the diagonal matrix with the sum of rows of $\mathbf{P}$: $\mathbf{W}_S$=diag($\sum_{ij} w_{ij} p_{ijk}$)=diag(p_{++k}).
Let $\mathbf{W}_C$ be the diagonal matrix with the sum of columns of $\mathbf{P}$:
$\mathbf{W}_C$=diag($\sum_k w_{ij} p_{ijk}$)=diag(w_{ij}).
First define

$$\overline{\mathbf{P}} = \mathbf{W}_S^{1/2}\left(\mathbf{W}_S^{-1}\mathbf{P}\mathbf{W}_C^{-1}\right)\mathbf{W}_C^{1/2}$$

and

$$\hat{\mathbf{P}} = \mathbf{W}_S^{1/2}\mathbf{Z}\left(\mathbf{Z}^t\mathbf{W}_S\mathbf{Z}\right)^{-1}\mathbf{Z}^t\mathbf{W}_S^{1/2}\overline{\mathbf{P}}\,.$$

CCA on $\mathbf{P}$ and $\mathbf{Z}$ is the generalized singular value decomposition (GSVD) of ($\hat{\mathbf{P}}^t, \mathbf{I}_{rm}, \mathbf{I}_S$) :

$$\hat{\mathbf{P}}\hat{\mathbf{P}}^t = \mathbf{V}\boldsymbol{\Psi}\mathbf{V}^t\,.$$

Link with DPCoA:

Let $\mathbf{P}_C = \mathbf{P}\mathbf{W}_C^{-1} = \left(p_{ijk}\right)$. Consider the GSVD of ($\mathbf{Z}$, $\mathbf{W}_S$, $\mathbf{I}_n$):

$$\mathbf{Z}^t\mathbf{W}_S\mathbf{Z} = \mathbf{U}\boldsymbol{\Lambda}\mathbf{U}^t\,.$$

Consider $\mathbf{X} = \mathbf{Z}\mathbf{U}\boldsymbol{\Lambda}^{-1/2}$. The Euclidean distances between the rows of $\mathbf{X}$ are Mahalanobis distances between the rows of $\mathbf{Z}$ weighted by $\mathbf{W}_S$. Let $\boldsymbol{\Delta}$ be a square matrix that contains these distances so that the species coordinates provided by the PCoA of $\boldsymbol{\Delta}$ weighted by $\mathbf{W}_S$ are in the rows of $\mathbf{X}$. In the space of DPCoA, the communities are positioned at

$$\mathbf{Y}_C = \mathbf{P}_C^t\mathbf{X}$$

Matrix $\hat{\mathbf{P}}$ can be rewritten as

$$\hat{\mathbf{P}} = \mathbf{W}_S^{1/2}\mathbf{Z}\left(\mathbf{Z}^t\mathbf{W}_S\mathbf{Z}\right)^{-1}\mathbf{Z}^t\mathbf{W}_S^{1/2}\mathbf{W}_S^{1/2}\left(\mathbf{W}_S^{-1}\mathbf{P}\mathbf{W}_C^{-1}\right)\mathbf{W}_C^{1/2},$$

$$\Leftrightarrow \hat{\mathbf{P}} = \mathbf{W}_S^{1/2}\mathbf{Z}\mathbf{U}\mathbf{\Lambda}^{-1/2}\mathbf{\Lambda}^{-1/2}\mathbf{U}^t\mathbf{Z}^t\mathbf{P}_C\mathbf{W}_C^{1/2},$$

$$\Leftrightarrow \hat{\mathbf{P}} = \mathbf{W}_S^{1/2}\mathbf{X}\mathbf{Y}_C^t\mathbf{W}_C^{1/2}.$$

The GSVD of $\hat{\mathbf{P}}\hat{\mathbf{P}}^t$ is

$$\hat{\mathbf{P}}\hat{\mathbf{P}}^t = \mathbf{W}_S^{1/2}\mathbf{X}\mathbf{Y}_C^t\mathbf{W}_C^{1/2}\mathbf{W}_C^{1/2}\mathbf{Y}_C\mathbf{X}^t\mathbf{W}_S^{1/2} = \mathbf{V}\mathbf{\Psi}\mathbf{V}^t,$$

$$\Leftrightarrow \mathbf{Y}_C^t\mathbf{W}_C\mathbf{Y}_C = \mathbf{X}^t\mathbf{W}_S^{1/2}\mathbf{V}\mathbf{\Psi}\mathbf{V}^t\mathbf{W}_S^{1/2}\mathbf{X}.$$

Consider $\mathbf{V}_* = \mathbf{X}^t\mathbf{W}_S^{1/2}\mathbf{V}$, then

$$\mathbf{Y}_C^t\mathbf{W}_C\mathbf{Y}_C = \mathbf{V}_*\mathbf{\Psi}\mathbf{V}_*^t,$$

which is the GSVD of $(\mathbf{Y}_C, \mathbf{W}_C, \mathbf{I}_v)$ and thus DPCoA of $\mathbf{P}_C$ and $\mathbf{\Delta}$. DPCoA on $\mathbf{P}_C$ and $\mathbf{\Delta}$ is thus equal to CCA on **P** and **Z**. The coordinates of the communities are in

$$\begin{aligned}\mathbf{Y}_C\mathbf{V}_* &= \mathbf{W}_C^{-1}\mathbf{P}^t\mathbf{X}\mathbf{X}^t\mathbf{W}_S^{1/2}\mathbf{V}\\ &= \mathbf{W}_C^{-1}\mathbf{P}^t\mathbf{Z}\left(\mathbf{Z}^t\mathbf{W}_S\mathbf{Z}\right)^{-1}\mathbf{Z}^t\mathbf{W}_S^{1/2}\mathbf{V},\end{aligned}$$

and those of the species are in

$$\begin{aligned}\mathbf{X}\mathbf{V}_* &= \mathbf{X}\mathbf{X}^t\mathbf{W}_S^{1/2}\mathbf{V}\\ &= \mathbf{Z}\left(\mathbf{Z}^t\mathbf{W}_S\mathbf{Z}\right)^{-1}\mathbf{Z}^t\mathbf{W}_S^{1/2}\mathbf{V}.\end{aligned}$$

CONNECTION BETWEEN DPCOA VERSION 1 AND PARTIAL CANONICAL CORRESPONDENCE ANALYSIS, FOUCART'S ANALYSIS AND PARTIAL NON-SYMETRIC CORRESPONDENCE ANALYSIS

When species are equidistant, the crossed-DPCoA reduces to the analysis of data on species relative abundances or presence/absence data. In this case, the crossed-DPCoA provides an alternative to partial canonical correspondence analysis [2], to Foucart's analysis [3] based on correspondence analysis and to the partial non-symmetric correspondence analysis [4].

Let **P** be a matrix of nonnegative values that sum to 1: $\mathbf{1}_S^t\mathbf{P}\mathbf{1}_{rm} = 1$. Consider that the columns of **P** are communities defined by two crossed factors A with *r* levels and B with *m* levels. We will consider hereafter the $S \times rm$ matrix $\mathbf{P} = \left(p_{ijk}w_{ij}\right)$, where p_{ijk} is the proportion of species *k* within community *ij* and w_{ij} is a weight attributed to community *ij* (see Text S1).

Ter Braak [2] popularized the partial canonical correspondence analysis as follows.

Let $\mathbf{U}_{\mathrm{A}}$ be the matrix with communities as rows and levels of factor A as columns. The entry at the ith row and j^{th} column contains 1 if the community i is associated with the jth level of factor A and 0 otherwise. This matrix is the disjunctive matrix associated with factor A.
Let $\mathbf{U}_{\mathrm{B}}$ be the matrix with communities as rows and levels of factor B as columns. The entry at the ith row and j^{th} column contains 1 if the community i is associated with the jth level of factor B and 0 otherwise. This matrix is the disjunctive matrix associated with factor B.
As in Text S2, the projector on the orthogonal complement of the subspace generated by $\mathbf{U}_{\mathrm{B}}$ is

$$\mathbf{P}_{\mathbf{U}_{\mathrm{B}}}^{\perp} = \mathbf{I}_{rm} - \mathbf{U}_{\mathrm{B}}\left(\mathbf{U}_{\mathrm{B}}^{t}\mathbf{W}_{\mathrm{C}}\mathbf{U}_{\mathrm{B}}\right)^{-1}\mathbf{U}_{\mathrm{B}}^{t}\mathbf{W}_{\mathrm{C}}$$

the projector on the subspace generated by $\mathbf{U}_{\mathrm{A}}$ is

$$\mathbf{P}_{\mathbf{U}_{\mathrm{A}}} = \mathbf{U}_{\mathrm{A}}\left(\mathbf{U}_{\mathrm{A}}^{t}\mathbf{W}_{\mathrm{C}}\mathbf{U}_{\mathrm{A}}\right)^{-1}\mathbf{U}_{\mathrm{A}}^{t}\mathbf{W}_{\mathrm{C}}$$

Let (A) be the space generated by $\mathbf{U}_{\mathrm{A}}$ and $(\mathrm{A})^{\perp}$ its complements. Similarly (B) is the space generated by $\mathbf{U}_{\mathrm{B}}$ and $(\mathrm{B})^{\perp}$ its complement. Let (A+B) be the space generated by $\mathbf{U}_{\mathrm{A+B}}$, where $\mathbf{U}_{\mathrm{A+B}}$ is the matrix with communities as rows obtained as follows: the first column contains only ones, the next columns are all but one column of $\mathbf{U}_{\mathrm{A}}$ and the last columns contain all but one column of $\mathbf{U}_{\mathrm{B}}$. In the case of orthogonal factors, $(\mathrm{A+B}) = (\mathrm{A}) \oplus (\mathrm{B})$ so that the orthogonal complement of (B) in (A+B) is (A). When the two factors are not orthogonal then $(\mathrm{A+B}) = (\mathrm{A/B}) \oplus (\mathrm{B})$. The subspace of (A+B) named (A/B) is generated by the matrix $\mathbf{P}_{\mathbf{U}_{\mathrm{B}}}^{\perp}\mathbf{U}_{\mathrm{A}}$. The projector on this subspace is

$$\mathbf{P}_{(\mathrm{A/B})} = \mathbf{P}_{\mathbf{U}_{\mathrm{B}}}^{\perp}\mathbf{U}_{\mathrm{A}}\left(\mathbf{U}_{\mathrm{A}}^{t}\mathbf{W}_{\mathrm{C}}\mathbf{P}_{\mathbf{U}_{\mathrm{B}}}^{\perp}\mathbf{U}_{\mathrm{A}}\right)^{-1}\mathbf{U}_{\mathrm{A}}^{t}\mathbf{W}_{\mathrm{C}}\mathbf{P}_{\mathbf{U}_{\mathrm{B}}}^{\perp}$$

The partial canonical correspondence analysis is the principal component analysis of

$$\mathbf{P}_{(\mathrm{A/B})}\mathbf{W}_{\mathrm{C}}^{-1}\mathbf{P}^{t}\mathbf{W}_{\mathrm{S}}^{-1}$$

where the weights of the rows of $\mathbf{P}$ are defined in $\mathbf{W}_{\mathrm{S}}$ and the weights of its columns in $\mathbf{W}_{\mathrm{C}}$ (see page 1).

Consider a matrix of equidistances among the species, say, $\boldsymbol{\Delta} = \sqrt{2}\left(\mathbf{1}_{S}\mathbf{1}_{S}^{t} - \mathbf{I}_{S}\right)$. The multiplicative factor $\sqrt{2}$ leads to a total inertia associated with $\boldsymbol{\Delta}$ equal to the between-communities Gini-Simpson diversity.

The PCoA on $\boldsymbol{\Delta}$ weighted by $\mathbf{W}_{\mathrm{S}}$ is the centred PCA of $\mathbf{I}_{\mathrm{S}}$ weighted by $\mathbf{W}_{\mathrm{S}}$ i.e.:

$$\left(\mathbf{I}_{\mathrm{S}} - \mathbf{1}_{\mathrm{S}}\mathbf{1}_{\mathrm{S}}^{t}\mathbf{W}_{\mathrm{S}}\right)^{t}\mathbf{W}_{\mathrm{S}}\left(\mathbf{I}_{\mathrm{S}} - \mathbf{1}_{\mathrm{S}}\mathbf{1}_{\mathrm{S}}^{t}\mathbf{W}_{\mathrm{S}}\right) = \mathbf{U}\boldsymbol{\Lambda}\mathbf{U}^{t}.$$

The species coordinates are given by $\mathbf{X} = \left(\mathbf{I}_{\mathrm{S}} - \mathbf{1}_{\mathrm{S}}\mathbf{1}_{\mathrm{S}}^{t}\mathbf{W}_{\mathrm{S}}\right)\mathbf{U}$.
The communities are positioned in the space of DPCoA at

$$\mathbf{W}_{\mathrm{C}}^{-1}\mathbf{P}^{t}\mathbf{X}$$

In crossed-DPCoA version 1 these coordinates are projected on $\mathbf{P}_{(\mathrm{A/B})}$ (see Text S2 for details) leading to new coordinates for the levels of factor A: $\mathbf{P}_{(\mathrm{A/B})}\mathbf{W}_{\mathrm{C}}^{-1}\mathbf{P}^{t}\mathbf{X}$. The principal axes of these newly defined points are obtained by the principal component analysis of

$$\mathbf{P}_{(\mathrm{A/B})}\mathbf{W}_{\mathrm{C}}^{-1}\mathbf{P}^{t}\mathbf{X}$$

where the weights of the rows are defined in $\mathbf{W}_C$ and the weights of the columns in $\mathbf{I}_v$.

This corresponds to

$$\mathbf{P}_{(A/B)}\mathbf{W}_C^{-1}\mathbf{P}^t\mathbf{X}\mathbf{X}^t\mathbf{P}\mathbf{W}_C^{-1}\mathbf{P}_{(A/B)} = \mathbf{P}_{(A/B)}\mathbf{W}_C^{-1}\mathbf{P}^t\left(\mathbf{I}_S - \mathbf{1}_S\mathbf{1}_S^t\mathbf{W}_S\right)^t\mathbf{W}_S\left(\mathbf{I}_S - \mathbf{1}_S\mathbf{1}_S^t\mathbf{W}_S\right)\mathbf{P}\mathbf{W}_C^{-1}\mathbf{P}_{(A/B)}$$

and thus to the principal component analysis of

$$\mathbf{P}_{(A/B)}\mathbf{W}_C^{-1}\mathbf{P}^t\left(\mathbf{I}_S - \mathbf{1}_S\mathbf{1}_S^t\mathbf{W}_S\right)^t$$

where the weights of the rows of **P** are defined in $\mathbf{W}_C$ and the weights of the columns in $\mathbf{W}_S$.

Lauro & Balbi [4] developed a partial NSCA, where the relative abundances of species within each community are compared to their average relative abundances over all levels of factor A but per level of factor B. The analysis simplifies to the principal component analysis of

$$\mathbf{P}_B^{\perp}\mathbf{W}_C^{-1}\mathbf{P}^t$$

where the weights of the rows are defined in $\mathbf{W}_C$ and the weights of the columns in $\mathbf{I}_S$ (the identity matrix). It corresponds to a within-class ***non-symmetric*** correspondence analysis in comparison with the within-class correspondence analysis which is the principal component analysis of

$$\mathbf{P}_B^{\perp}\mathbf{W}_C^{-1}\mathbf{P}^t\mathbf{W}_S^{-1}$$

where the weights of the rows are defined in $\mathbf{W}_C$ and the weights of the columns in $\mathbf{W}_S$ (see [5] and references therein).

With Foucart's analysis, **P** is separated into submatrices per level of factor B: $\mathbf{P}_1$, $\mathbf{P}_2$, …, $\mathbf{P}_m$. Each matrix is divided by the sum of all of its values and a reference matrix is defined as the mean of all submatrices. The average effect of factor A is evaluated by this reference matrix. In addition all submatrices are projected on the analysis of the reference matrix to evaluate the differences between the average matrix and the submatrices. The effect of factor A is thus averaged over all levels of factor B (See [3] for details).

Each of these approaches (partial canonical correspondence analysis, partial non-symmetrical correspondence analysis, Foucart's analysis and crossed-DPCoA) thus treats differently the case where species are set to be equidistant. The advantage of the general form of the crossed-DPCoA (with any matrix $\mathbf{\Delta}$) is that any attribute (e.g. taxonomic, phylogenetic and functional) of species can also be incorporated into the analysis.

1. Pavoine S, Dufour AB, Chessel D (2004) From dissimilarities among species to dissimilarities among communities: a double principal coordinate analysis. J Theor Biol 228: 523-537.
2. Ter Braak CJF (1987) Unimodal models to relate species to environment. Agricultural Mathematics Groups. Wageningen, the Netherlands: Box, Box 100, NL-6700 AC. pp. 83-89.
3. Pavoine S, Blondel J, Baguette M, Chessel D (2007) A new technique for ordering asymmetrical three-dimensional data sets in ecology. Ecology 88: 512-523.
4. Lauro C, Balbi S (1999) The analysis of structured qualitative data. Applied Stochastic Models and Data Analysis 15: 1-27.
5. Yoccoz N, Chessel D (1988) Ordination sous contraintes de relevés d'avifaune : élimination d'effets dans un plan d'observations à deux facteurs. Compte rendu hebdomadaire des séances de l'Académie des sciences Paris, D III: 307 : 189-194.
